# Supplementary material for: Sub-Nanomolar Detection and Discrimination of Microcystin Congeners Using Aerolysin Nanopores
Source: ACS Nano. 2026 Jul 31;20(31):21857–71. doi: 10.1021/acsnano.6c05413 (PMC13472326; doi:10.1021/acsnano.6c05413)
Supplement: Supplementary file 1 [file nn6c05413_si_001.pdf]

Supplementary Information for

Sub-Nanomolar Detection and Discrimination of Microcystin Congeners Using Aerolysin Nanopores

Alissa Agerova[1,2], Juan Francisco Bada Juarez[1]*, Louis W. Perrin[3], Luciano A. Abriata[1], Maria J. Marcaida[1], Anna Carratalà[2], Nora Selmani[4], Stéphanie Barbier[4], Fereidoun Khajehnouri[4], Giordano Vassalli[5], Elisabeth M. L. Janssen[6], Chan Cao[3], Tamar Kohn[2]*, Matteo Dal Peraro[1]*

[1] Institute of Bioengineering, School of Life Science, École Polytechnique Fédérale de Lausanne (EPFL), 1015 Lausanne, Switzerland

[2] Laboratory of Environmental Virology, School of Architecture, Civil and Environmental Engineering (ENAC), École Polytechnique Fédérale de Lausanne (EPFL), 1015 Lausanne, Switzerland

[3] Department of Inorganic and Analytical Chemistry, School of Chemistry and Biochemistry, University of Geneva, 1205 Geneva, Switzerland

[4] Service de l'eau, Ville de Lausanne, Direction de la sécurité et de l'économie, 1001 Lausanne, Switzerland

[5] Centro di competenze sull'acqua, CCA SA, 6830 Chiasso, Switzerland

[6] Department of Environmental Chemistry, Swiss Federal Institute of Aquatic Science and Technology (EAWAG), 8600 Dübendorf, Switzerland

*Corresponding authors: juan.badajuarez@epfl.ch, tamar.kohn@epfl.ch, matteodalperaro@epfl.ch

Materials

All reagents used were of analytical-grade quality. The stock solutions of microcystins (Enzo Life Sciences, Farmingdale, NY, USA) at 1 mM (MC-LR, MC-RR), 200 µM (MC-YR, MC-LA, MC-LF, MC-LY, MC-LW) were dissolved in ultrapure water, and stored at −20 °C. Electrolyte solution was composed of 4M KCl (Sigma-Aldrich Chemie GmbH, Buchs, Switzerland), 10 mM Tris (Fischer, UK), 1.0 mM EDTA (Fisher, UK), and the pH was adjusted to 7.5. Prior to single-channel experiments, stock solutions of MCs at 200 nM in the respective electrolyte buffer were prepared.

Supplementary Figures

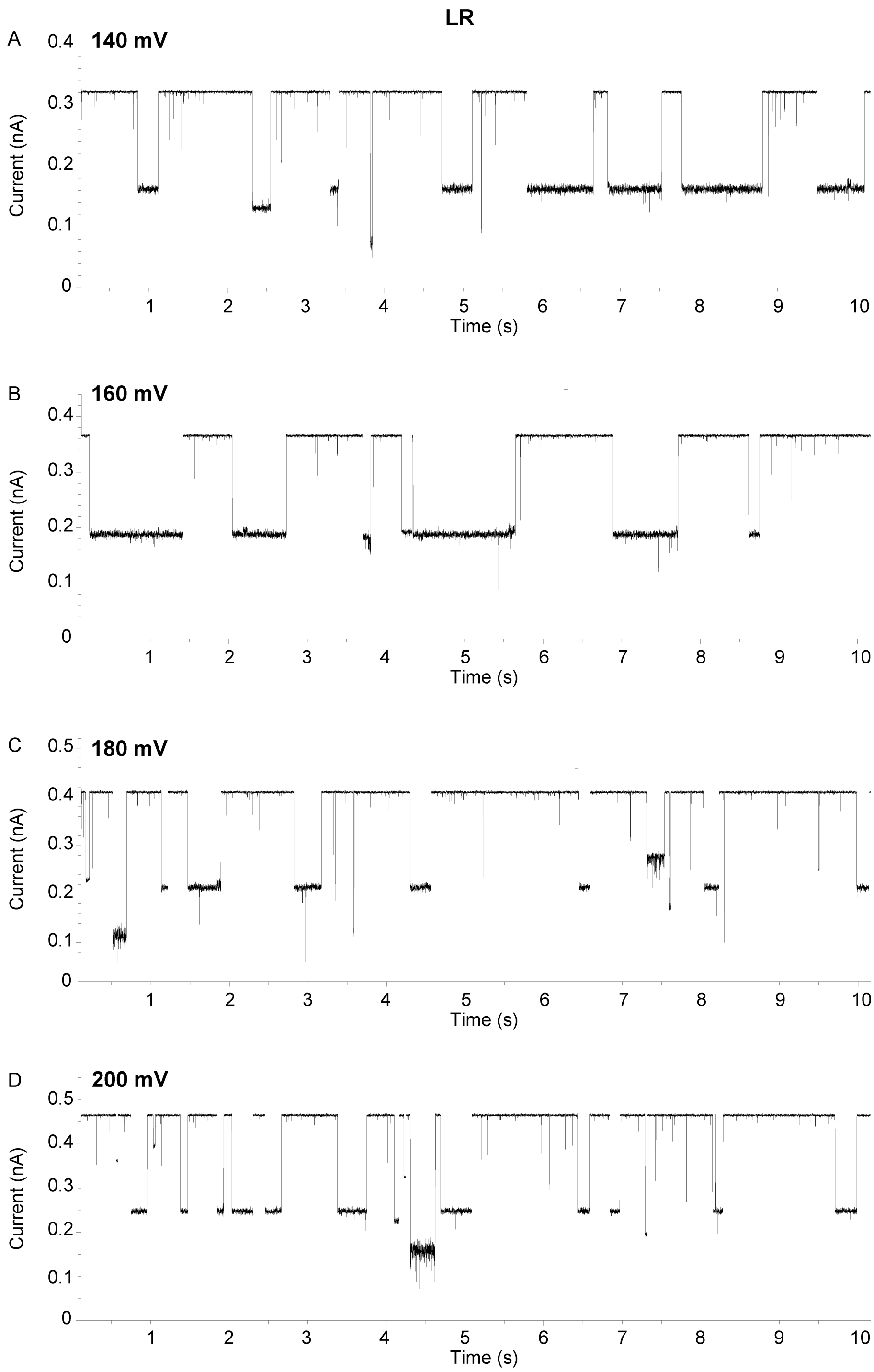

Figure S1. Raw current traces of MC-LR. Raw current traces at 140mV **(A)**, 160 mV **(B)**, 180 mV **(C)**, 200 mV **(D)** acquired in 4 M KCl, buffered with 10 mM Tris, 1.0 mM EDTA at pH 7.5; traces were filtered to 500 Hz. Increasing voltage results in shorter dwell times, as shown in the traces. Data extraction was performed as described in the Methods section.

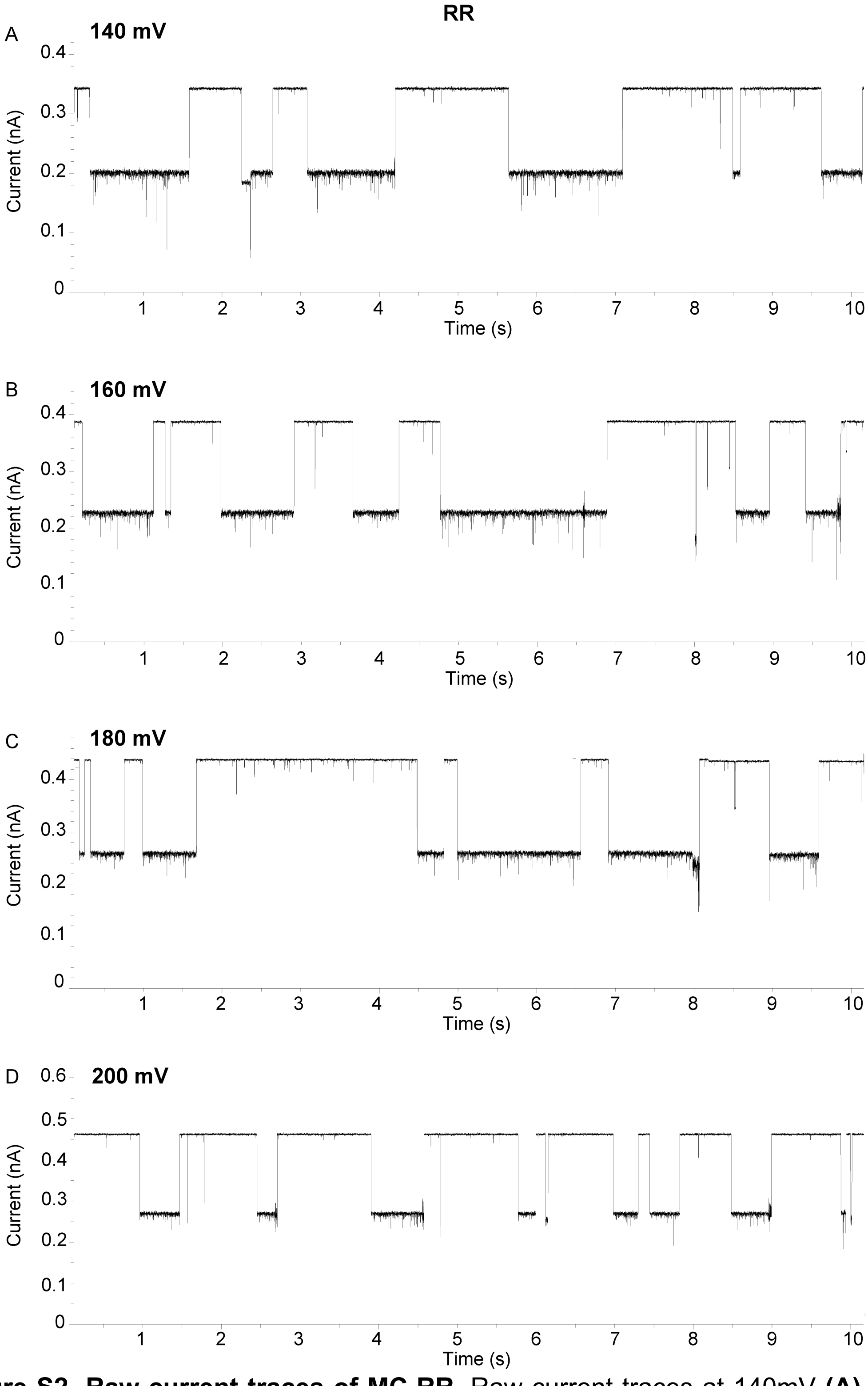

Figure S2. Raw current traces of MC-RR. Raw current traces at 140mV **(A)**, 160 mV **(B)**, 180 mV **(C)**, 200 mV **(D)** acquired in 4 M KCl, buffered with 10 mM Tris, 1.0 mM EDTA at pH 7.5; traces were filtered to 500 Hz. As the voltage increases, dwell times initially become longer

(up to 160 mV) but decrease from 180 mV. Data extraction was performed as described in the Methods section.

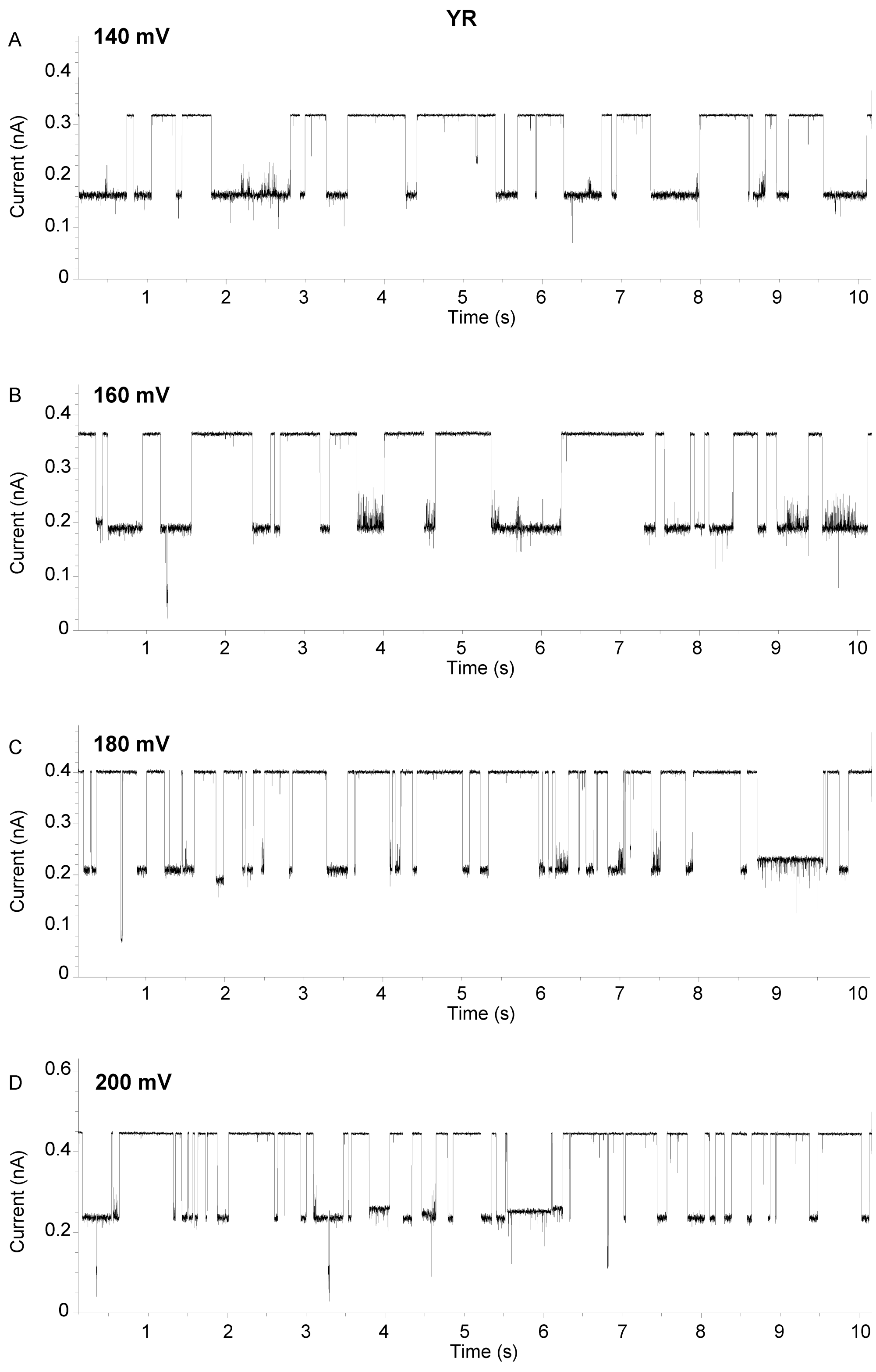

Figure S3. Raw current traces of MC-YR. Raw current traces at 140mV **(A)**, 160 mV **(B)**, 180 mV **(C)**, 200 mV **(D)** acquired in 4 M KCl, buffered with 10 mM Tris, 1.0 mM EDTA at pH 7.5; traces were filtered to 500 Hz. Increasing voltage results in shorter dwell times, as shown in the traces. Data extraction was performed as described in the Methods section.

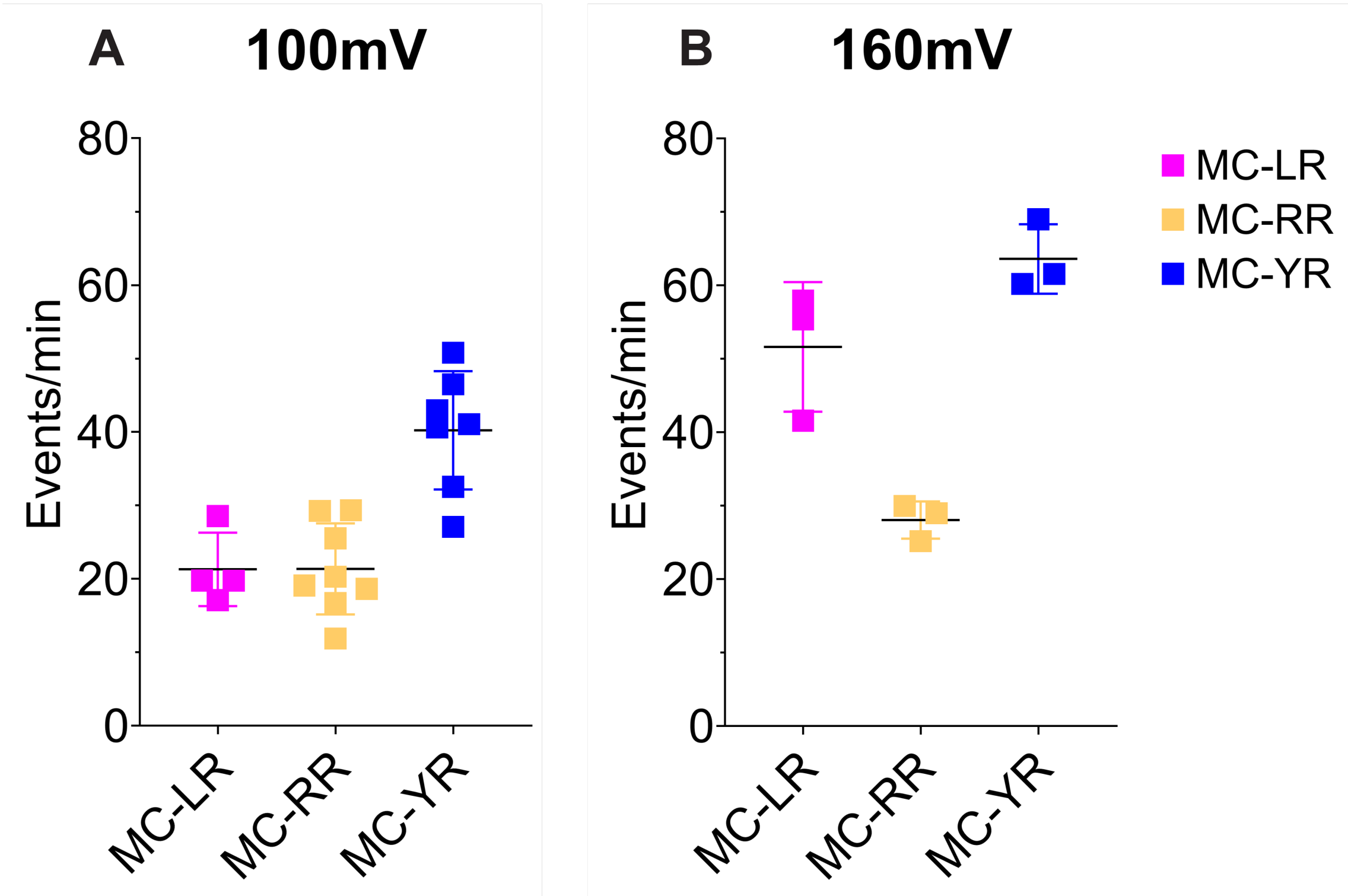

Events per minute	MC-LR	MC-RR	MC-YR
100 mV	21.3 ± 5.0	21.34 ± 6.19	40.22 ± 8.07
160 mV	51.62 ± 8.81	28.03 ± 2.53	63.58 ± 4.74

Figure S4. Event rates analysis of MC-LR, -RR, -YR congeners in individual experiments at 100 mV and 160 mV. Event rates (events/min) from single-pore recordings of each congener at 100 mV **(A)** and 160 mV **(B)**, where each square represents an individual pore. Mean values are shown below; 160 mV data were used to determine concentration ratios for uniform signal distribution in mixture experiments.

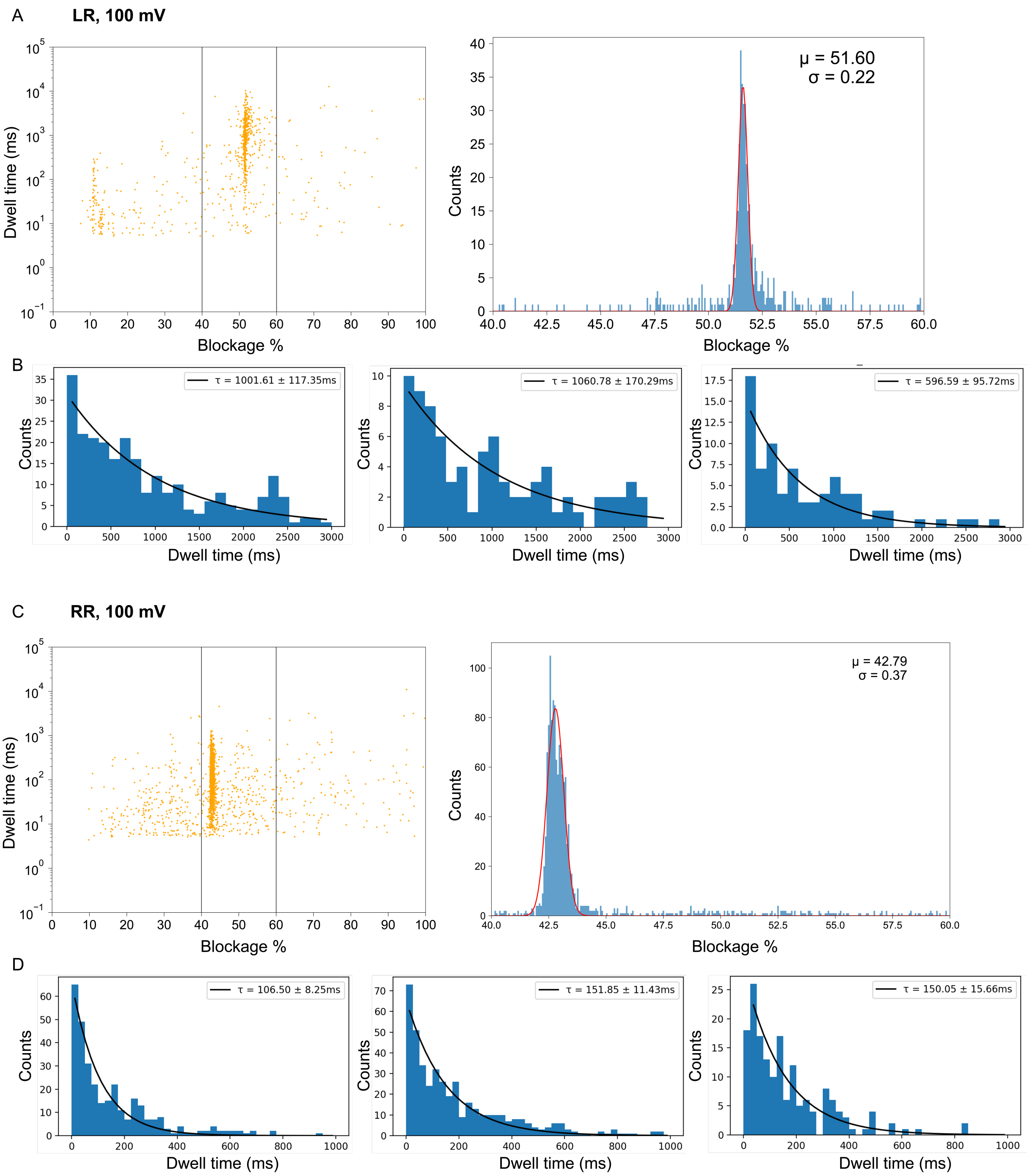

A
LR, 100 mV
Dwell time (ms)
Blockage %
Counts
μ = 51.60
σ = 0.22
B
τ = 1001.61 ± 117.35ms
τ = 1060.78 ± 170.29ms
τ = 596.59 ± 95.72ms
Dwell time (ms)
C
RR, 100 mV
μ = 42.79
σ = 0.37
D
τ = 106.50 ± 8.25ms
τ = 151.85 ± 11.43ms
τ = 150.05 ± 15.66ms

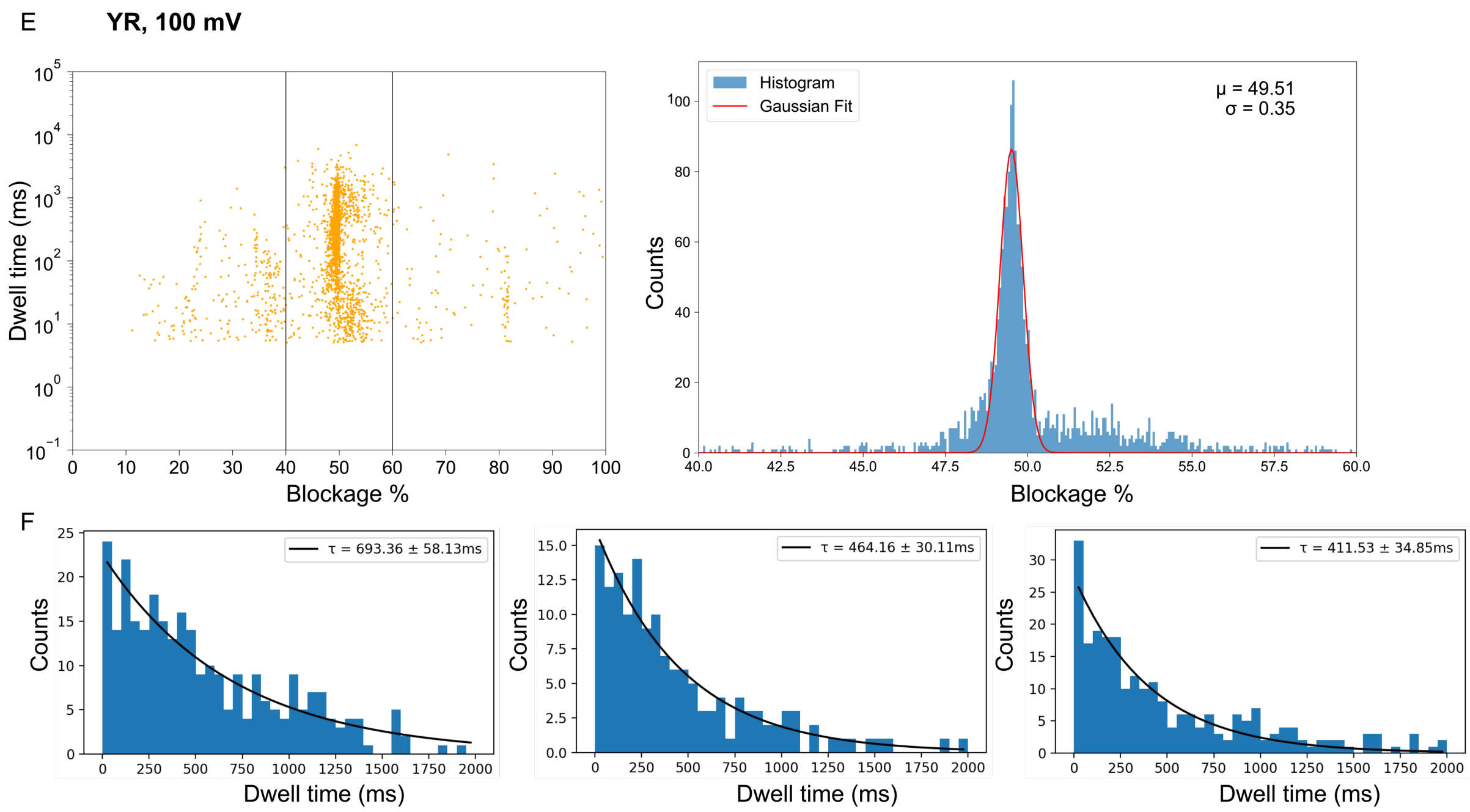

Figure S5. Event selection of MC-LR, -RR, -YR at 100 mV. (A, C, E) Overlay of scatterplots across at least 3 pore replicas produced by MC-LR, -RR, -YR interaction with WT AeL at 100 mV in individual experiments, respectively, event selection denoted by a box (left; 40 % - 60 %), and the respective overlay of current histograms produced by MC-LR, -RR, -YR at 100 mV (bin size = 300), respectively (right). The event selection window of 40 % - 60 % blockage was chosen based on the range where specific and reproducible events corresponding to the respective MC congeners consistently appeared, forming a well-defined population within this interval. Occasional events observed at lower or higher blockage levels were not consistently present across different pores and are attributed to transient bumping interactions or increased background noise under 4 M KCl conditions (lower blockage), as well as occasional pore clogging or self-blockage events (higher blockage). **(B, D, F)** Exponential fitting (bin size = 20) of MC-LR, -RR, -YR dwell times, respectively, per single pore, with 3 representative individual pore replicas illustrated. Data extraction was performed as described in the Methods.

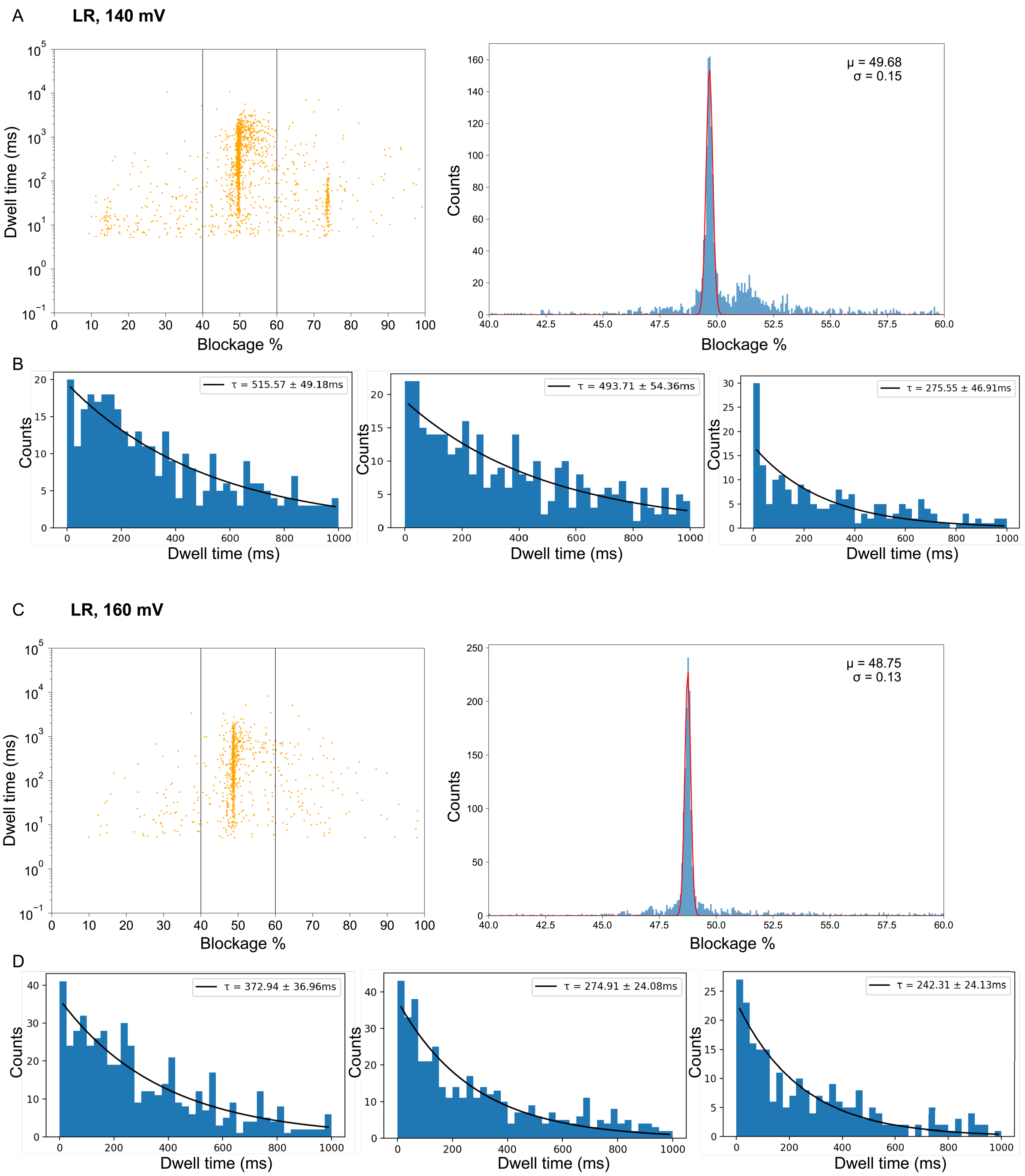

A
LR, 140 mV
Dwell time (ms)
Blockage %
Counts
μ = 49.68
σ = 0.15
B
τ = 515.57 ± 49.18ms
τ = 493.71 ± 54.36ms
τ = 275.55 ± 46.91ms
Counts
Dwell time (ms)
C
LR, 160 mV
Dwell time (ms)
Blockage %
Counts
μ = 48.75
σ = 0.13
D
τ = 372.94 ± 36.96ms
τ = 274.91 ± 24.08ms
τ = 242.31 ± 24.13ms
Counts
Dwell time (ms)

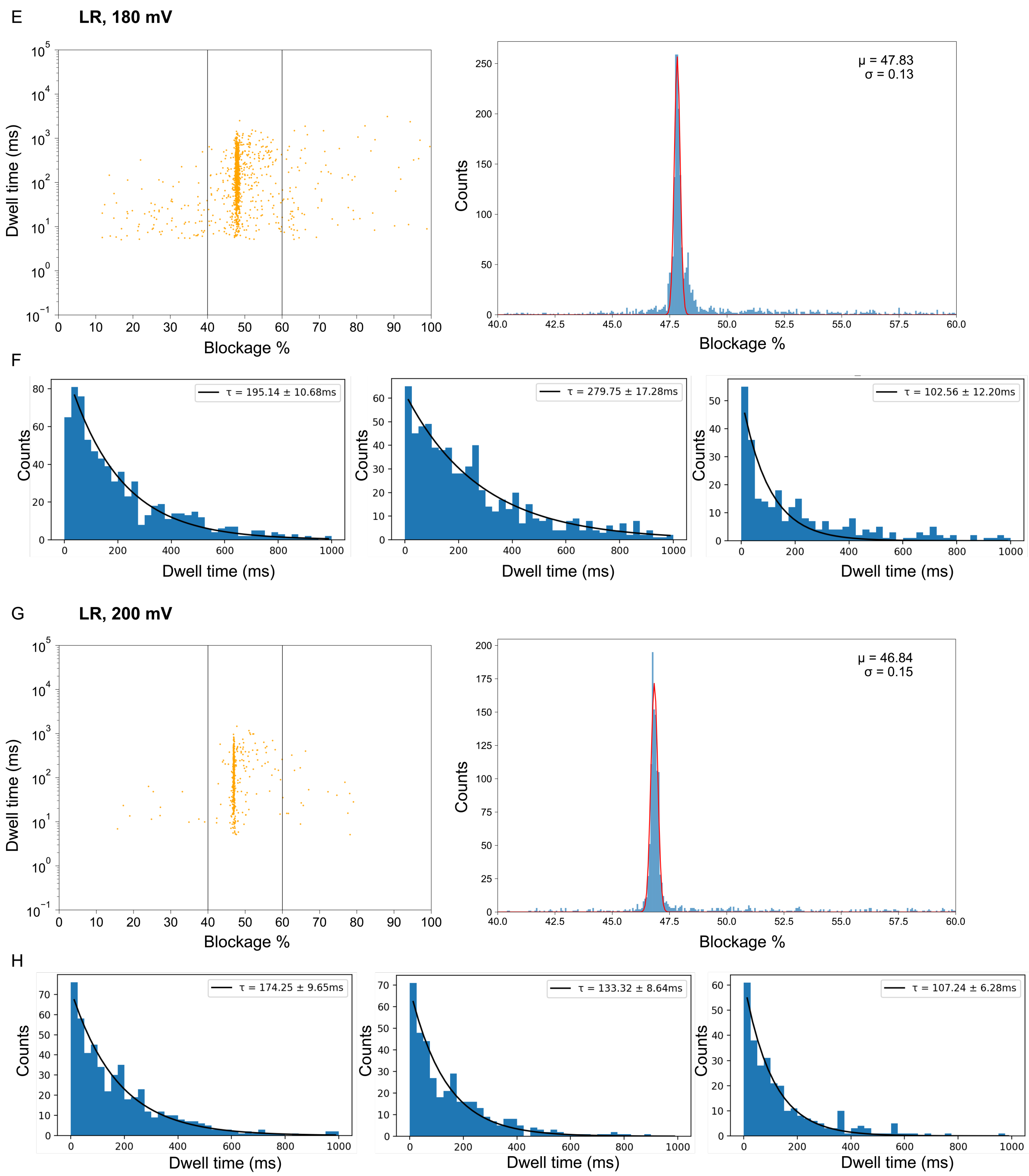

Figure S6. Event selection of MC-LR at 140 mV, 160 mV, 180 mV, 200 mV. (A, C, E, G) Overlay of scatterplots across at least 3 pore replicas, event selection denoted by a box (left; 40 % - 60 %), and the respective overlay of current histograms (bin size = 300) produced by MC-LR interaction with WT Ael at the specific voltage (right). **(B, D, F, H)** Exponential fitting (bin size = 20) of MC-LR dwell times per single pore, with 3 representative individual pore replicas illustrated. Data extraction was performed as described in the Methods.

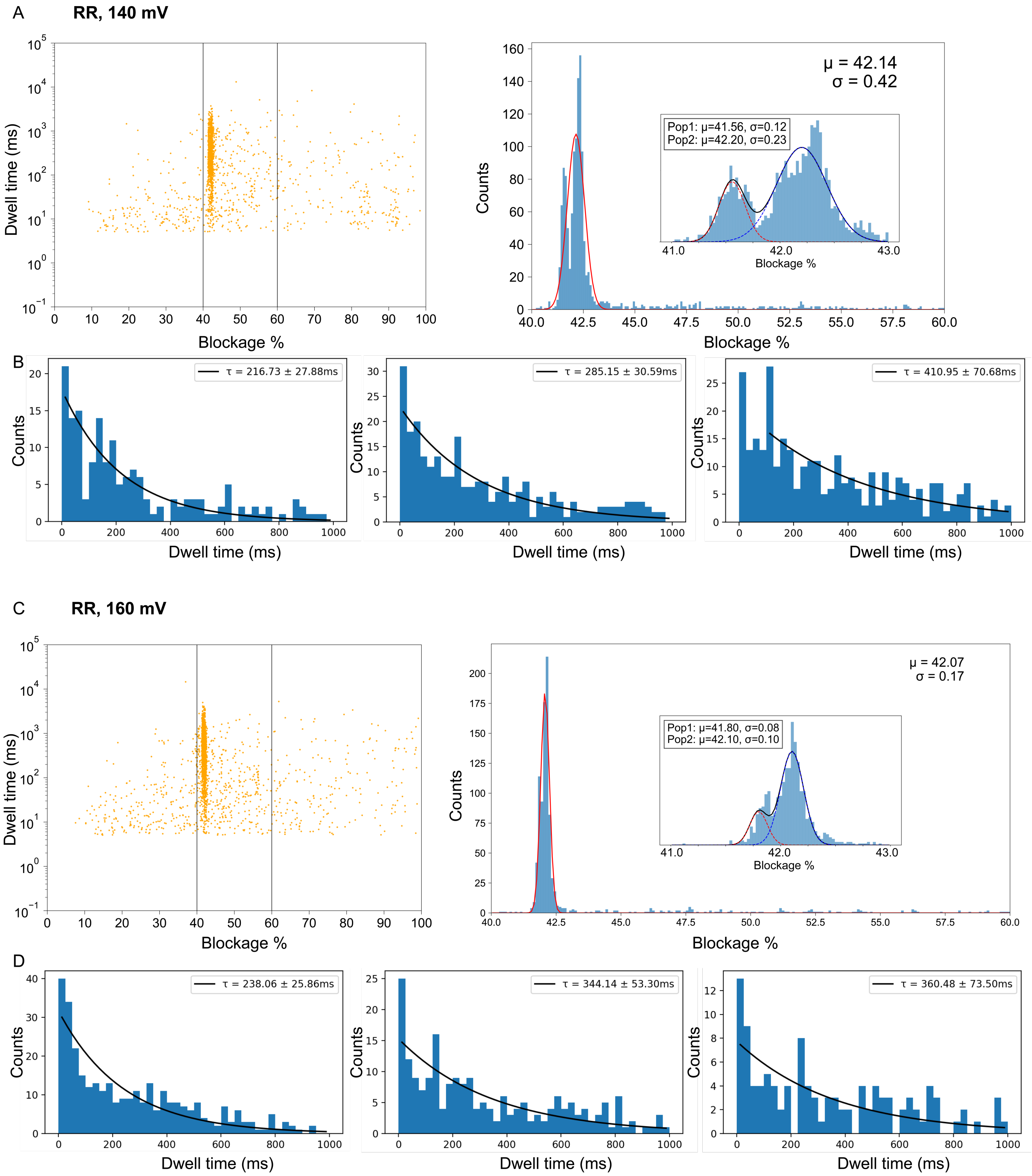

A
RR, 140 mV
Dwell time (ms)
Blockage %
Counts
μ = 42.14
σ = 0.42
Pop1: μ=41.56, σ=0.12
Pop2: μ=42.20, σ=0.23
B
τ = 216.73 ± 27.88ms
τ = 285.15 ± 30.59ms
τ = 410.95 ± 70.68ms
C
RR, 160 mV
μ = 42.07
σ = 0.17
Pop1: μ=41.80, σ=0.08
Pop2: μ=42.10, σ=0.10
D
τ = 238.06 ± 25.86ms
τ = 344.14 ± 53.30ms
τ = 360.48 ± 73.50ms

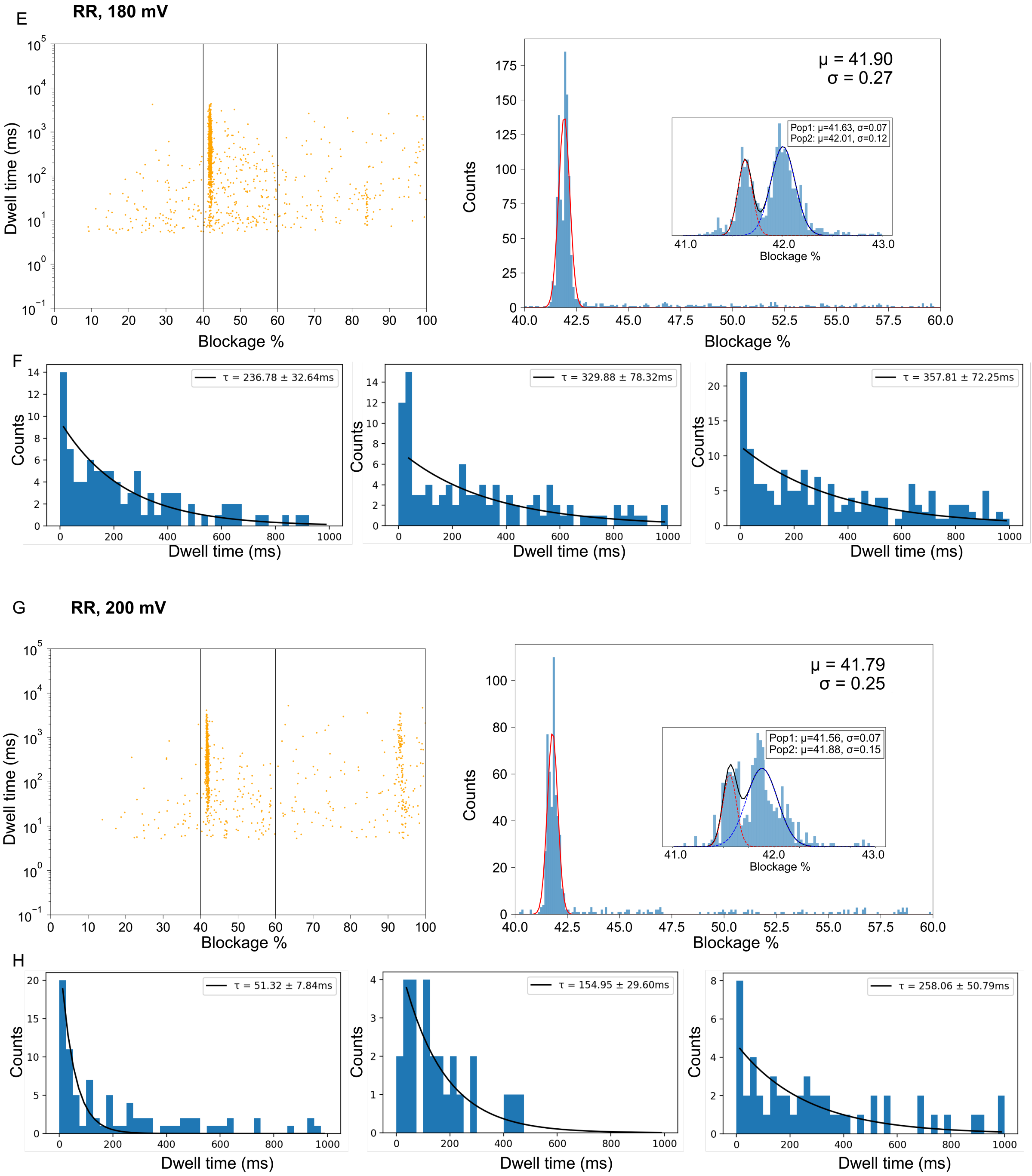

Figure S7. Event selection of MC-RR at 140 mV, 160 mV, 180 mV, 200 mV. (A, C, E, G) Overlay of scatterplots across at least 3 pore replicas, event selection denoted by a box (left; 40 % - 60 %), and the respective overlay of current histograms (bin size = 300 for single Gaussian fit; bin size = 75 for bimodal Gaussian fit) produced by MC-RR interaction with WT AeL at a specific voltage (right). **(B, D, F, H)** Exponential fitting (bin size = 20) of MC-RR dwell times per single pore, with 3 representative individual pore replicas illustrated. Data extraction was performed as described in the Methods.

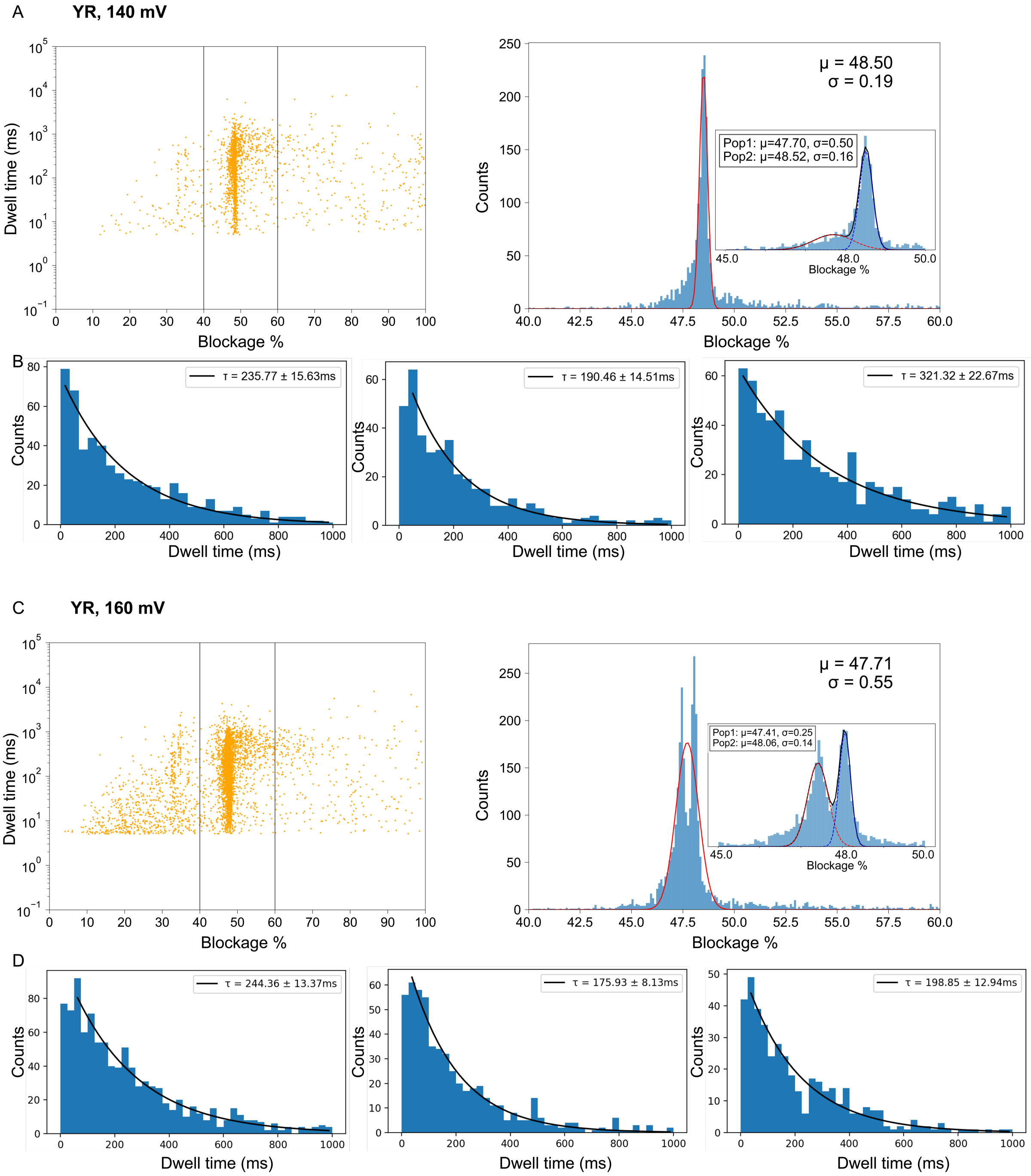
A
YR, 140 mV
Dwell time (ms)
Blockage %
Counts
μ = 48.50
σ = 0.19
Pop1: μ=47.70, σ=0.50
Pop2: μ=48.52, σ=0.16
B
τ = 235.77 ± 15.63ms
τ = 190.46 ± 14.51ms
τ = 321.32 ± 22.67ms
Dwell time (ms)
C
YR, 160 mV
μ = 47.71
σ = 0.55
Pop1: μ=47.41, σ=0.25
Pop2: μ=48.06, σ=0.14
D
τ = 244.36 ± 13.37ms
τ = 175.93 ± 8.13ms
τ = 198.85 ± 12.94ms

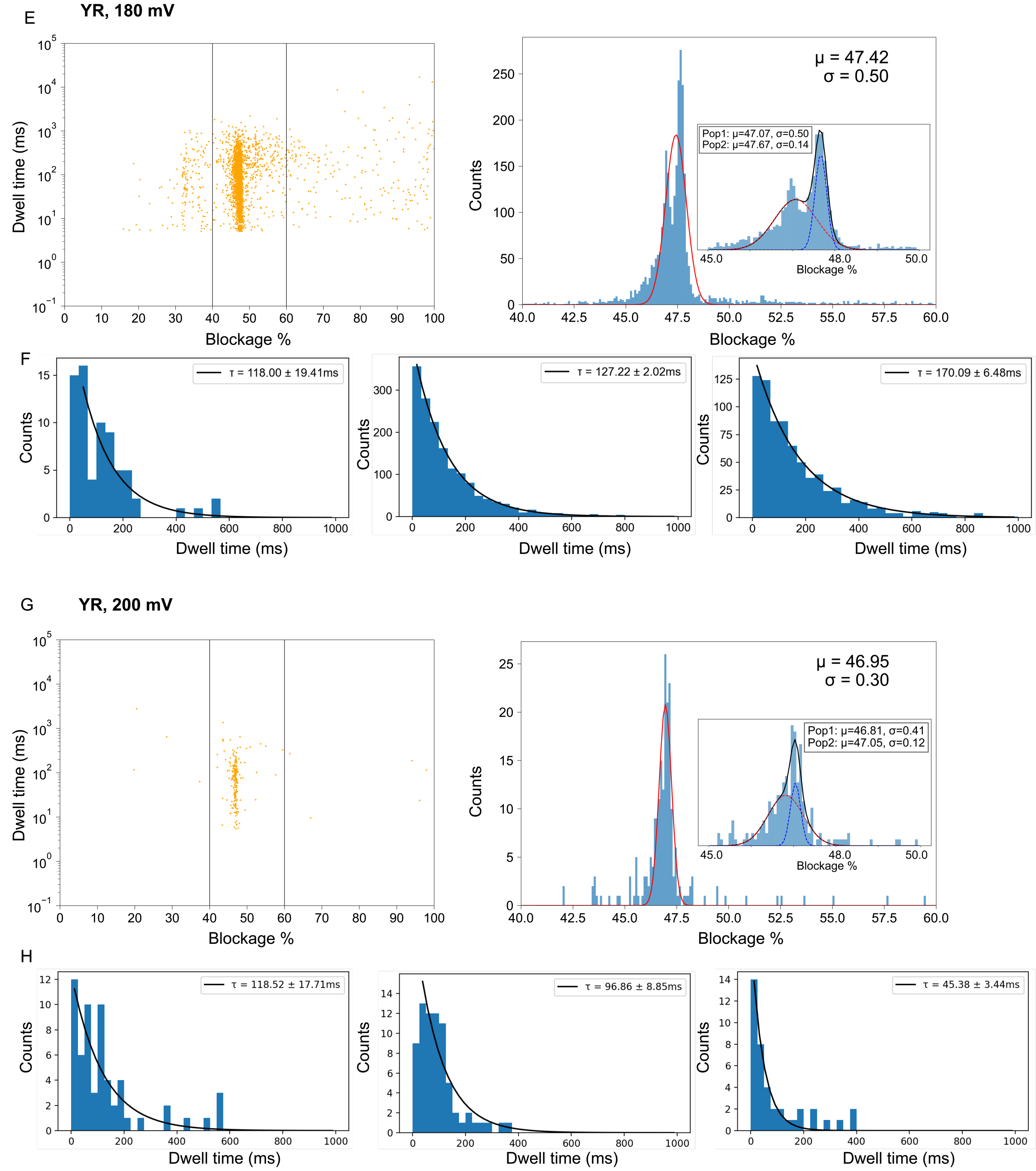

Figure S8. Event selection of MC-YR at 140 mV, 160 mV, 180 mV, 200 mV. (A, C, E, G) Overlay of scatterplots across at least 3 pore replicas, event selection denoted by a box (left; 40 % - 60 %), and the respective overlay of current histograms (bin size = 300 for single Gaussian fit; bin size = 75 for bimodal Gaussian fit) produced by MC-YR interaction with WT AeL at a specific voltage (right). **(B, D, F, H)** Exponential fitting (bin size = 20) of MC-YR dwell times per single pore, with 3 representative individual pore replicas illustrated. Data extraction was performed as in the Methods.

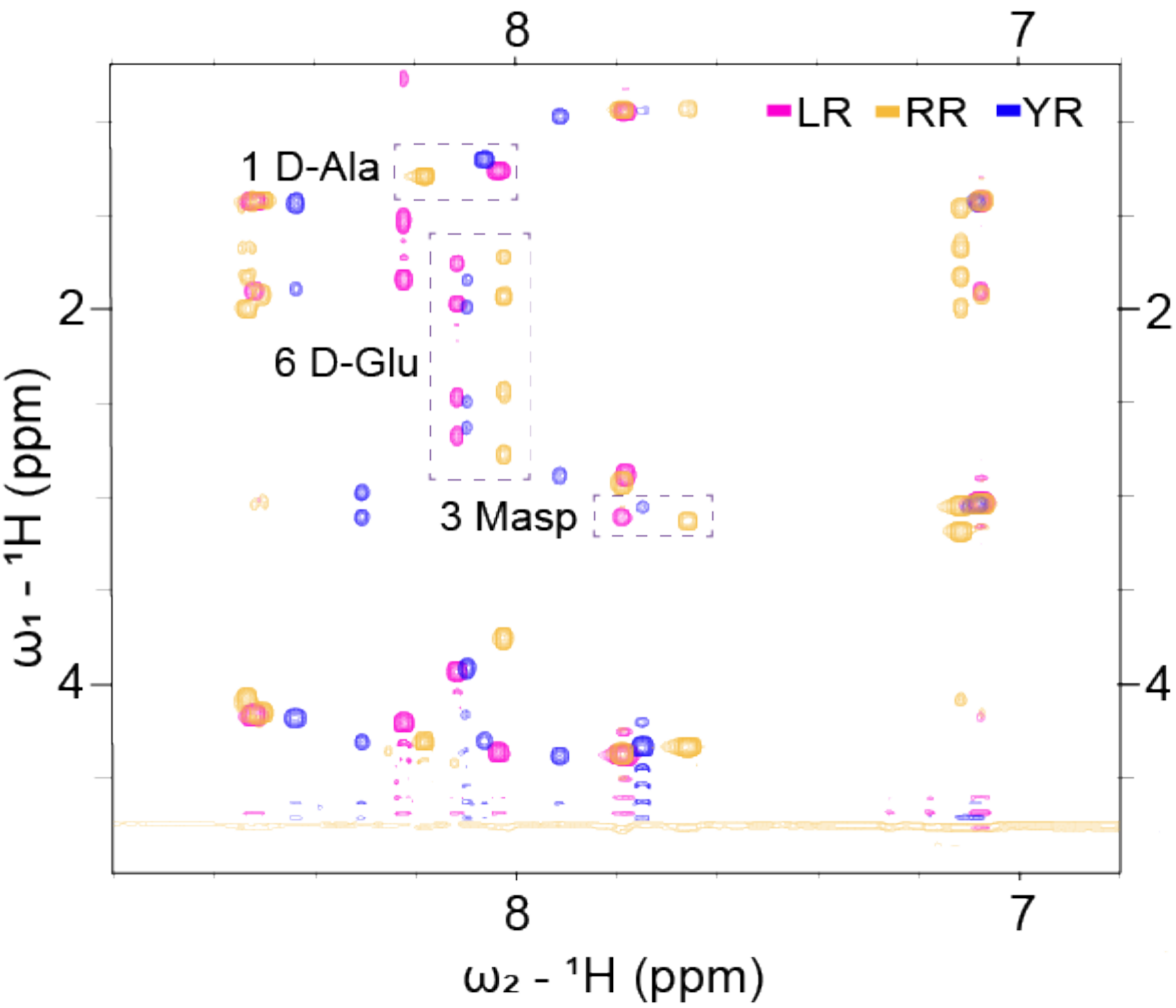

Figure S9. TOCSY patterns emanating from HN groups in $^{1}H,^{1}H$ TOCSY NMR spectra for MC-LR, -RR, -YR. Similarities and differences are highlighted with boxes and labelled with assignments adapted to water conditions from published assignments[1–3] assisted with NOESY and ^{13}C-HSQC spectra.

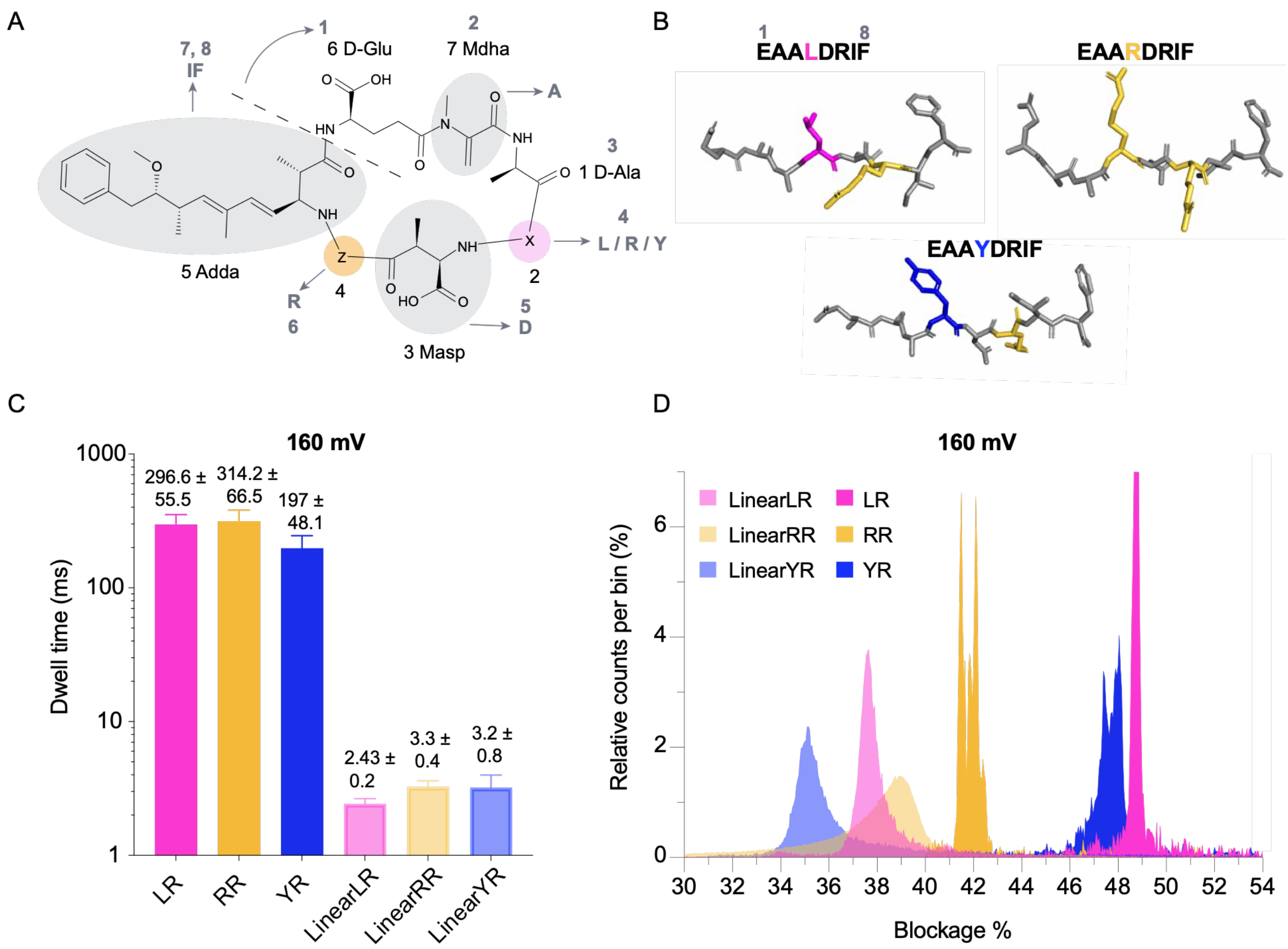

Figure S10. Linear MC-like peptides and their detection by the aerolysin nanopore. (A) Structure of MC, where non-proteinogenic residues are substituted with natural amino acid (highlighted in gray), Position 2 is a variable region in cyclic MC (highlighted in pink, being Position 4 in the linearMC). Chemical groups or residues are numbered, and their abbreviations are given. The total net charge in LinearLR (-1), LinearRR (0), LinearYR (0), matches the net charge of the respective cyclic MC. **(B)** Alphafold predicted structures of linear analogs. **(C)** Dwell time at 160 mV of MC and the respective linear analog in individual experiments. **(D)** Current blockage by linear and cyclic peptides at 160 mV in individual experiments. Data acquired at the experimental conditions of 4 M KCl, pH 7.4, 25°C, using WT AeL, 10 μM of linear analog, 50 nM cyclic MC.

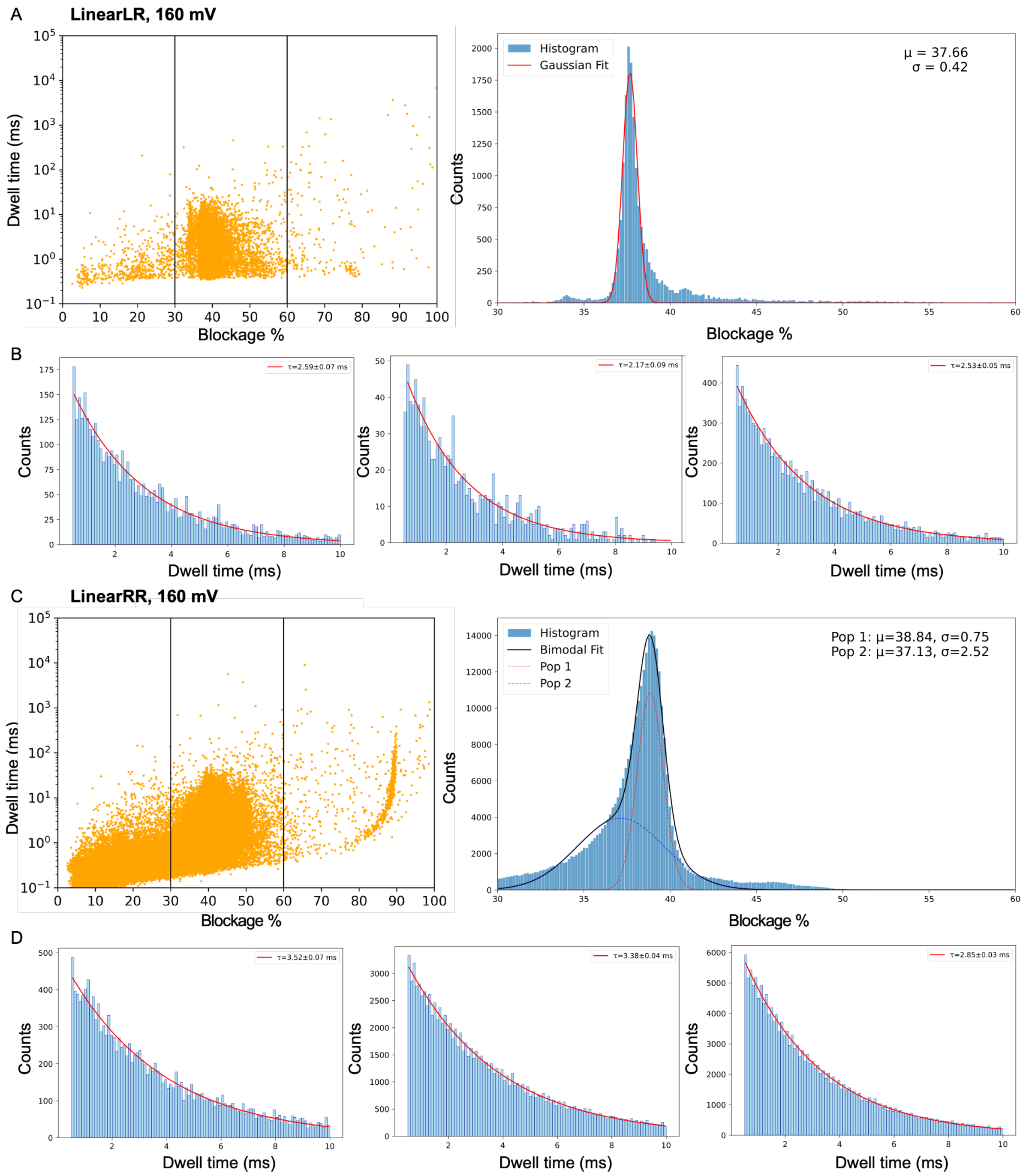

A
LinearLR, 160 mV
Dwell time (ms)
Blockage %
Histogram
Gaussian Fit
μ = 37.66
σ = 0.42
Counts
Blockage %
B
τ=2.59±0.07 ms
τ=2.17±0.09 ms
τ=2.53±0.05 ms
Counts
Dwell time (ms)
C
LinearRR, 160 mV
Dwell time (ms)
Blockage %
Histogram
Bimodal Fit
Pop 1
Pop 2
Pop 1: μ=38.84, σ=0.75
Pop 2: μ=37.13, σ=2.52
Counts
Blockage %
D
τ=3.52±0.07 ms
τ=3.38±0.04 ms
τ=2.85±0.03 ms
Counts
Dwell time (ms)

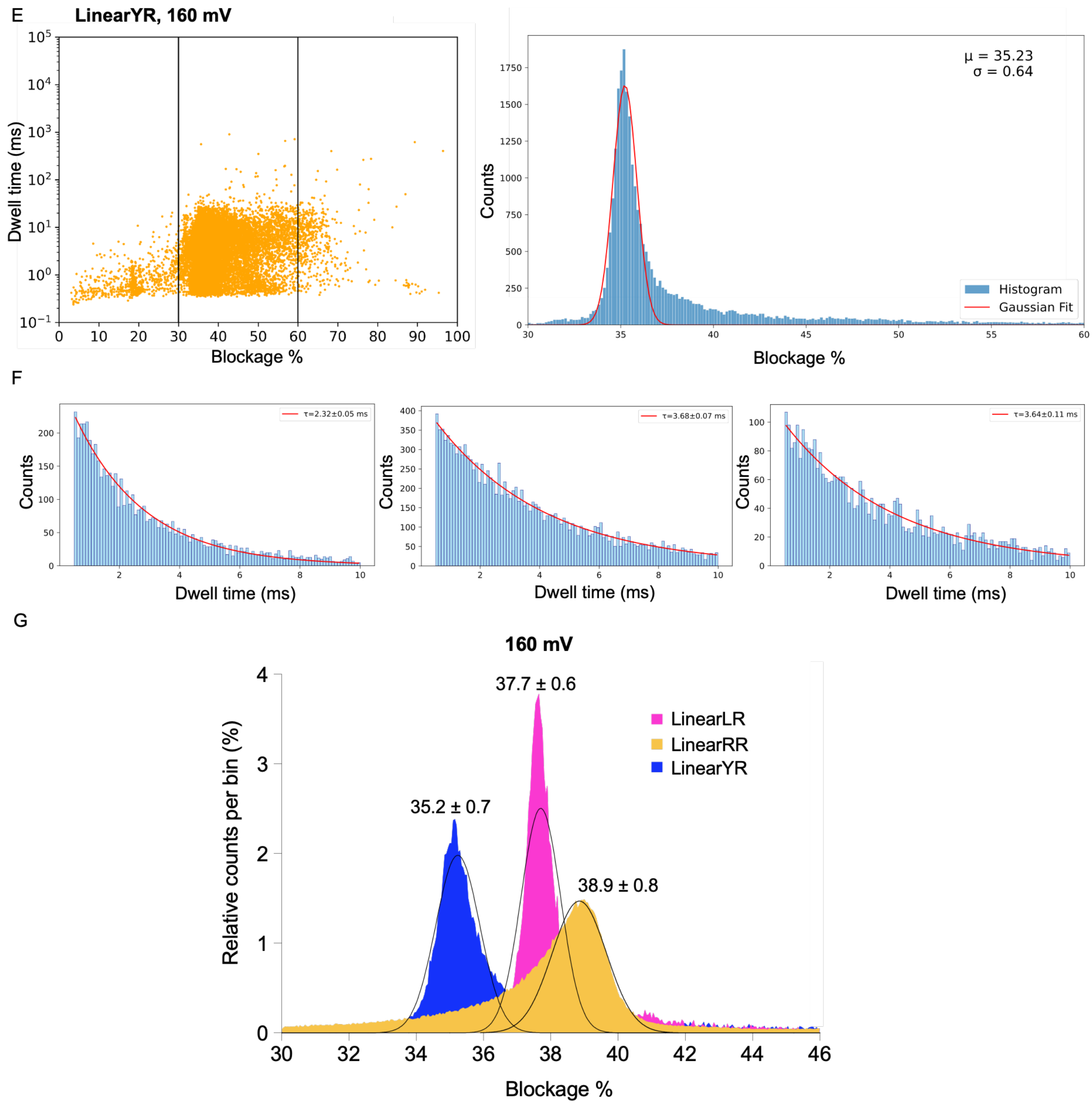

Figure S11. Event selection of LinearLR, -RR, -YR at 160 mV. (A, C, E) Overlay of scatterplots across at least 3 pore replicas produced by LinearLR, -RR, -YR interaction with WT AeL at 160 mV in individual experiments, respectively, event selection denoted by a box (left; 30 % - 60 %), and the respective overlay of current histograms produced by LinearLR, -RR, -YR at 160 mV (bin size = 200), respectively (right). The event selection window of 30 % - 60 % blockage was chosen based on the range where specific and reproducible events corresponding to the respective LinearMC consistently appeared, forming a well-defined population within this interval. Occasional events observed at lower or higher blockage levels were not consistently present across different pores and are attributed to transient bumping interactions or increased background noise under 4 M KCl conditions (lower blockage), as well as occasional pore clogging or self-blockage events (higher blockage). **(B, D, F)** Exponential fitting (bin size = 100) of LinearLR, -RR, -YR dwell times, respectively, per single pore, with 3 representative individual pore replicas illustrated. (G) Overlay of current blockage histograms at 160 mV with Gaussian fits distinguishing LinearLR (37.7±0.8), -RR (38.9±0.8), -YR (35.2±0.7), populations. Data extraction was performed as described in the Methods.

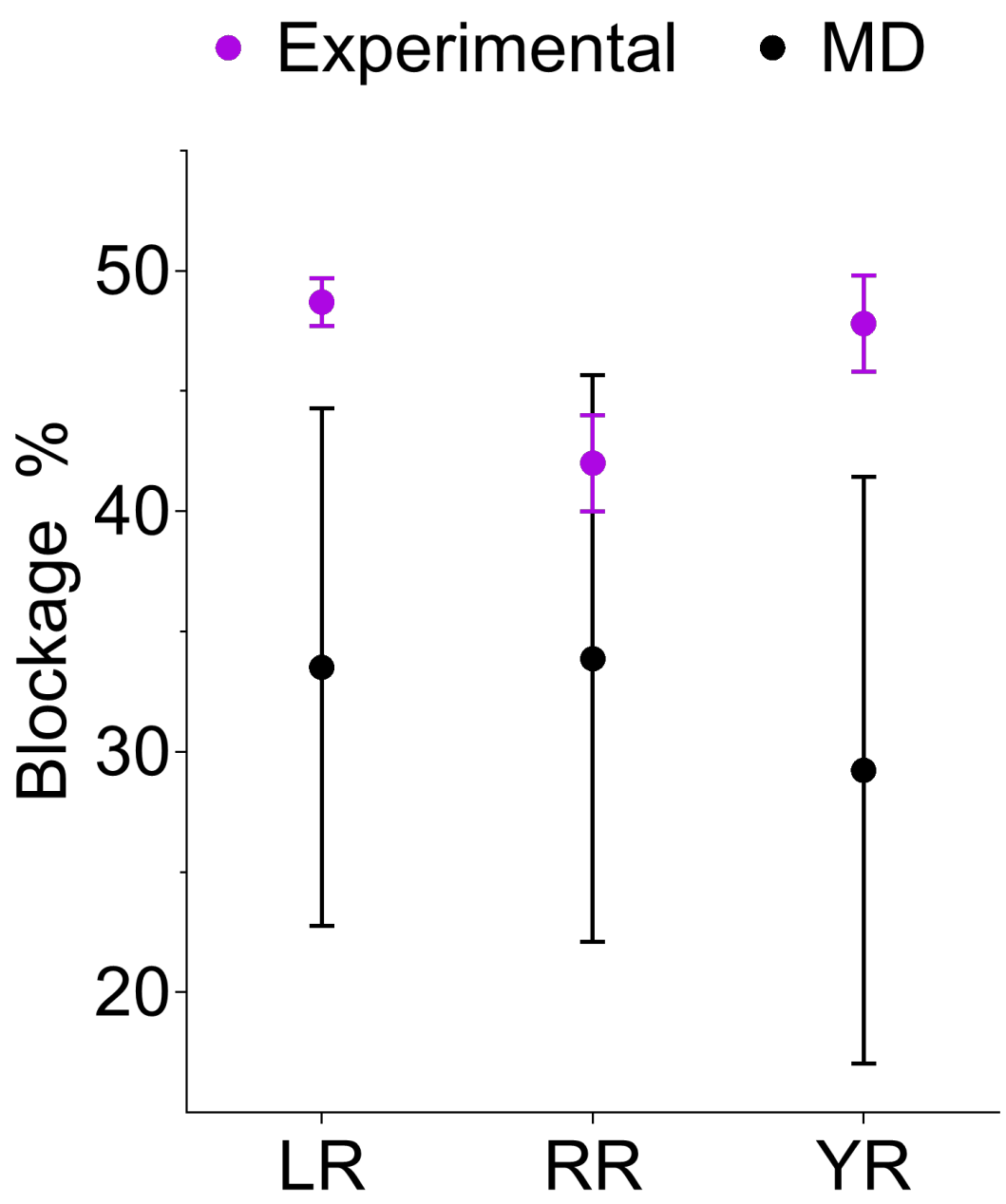

Figure S12. Comparison of experimental and MD results yielding from MC-LR, -RR, -YR blockage of WT AeL at 160 mV. MD simulations were performed as described in the Methods. Considering the significant standard deviation of the MD replicas, we conclude that MC-LR, -RR, and -YR behave the same within statistical significance, as determined by MD.

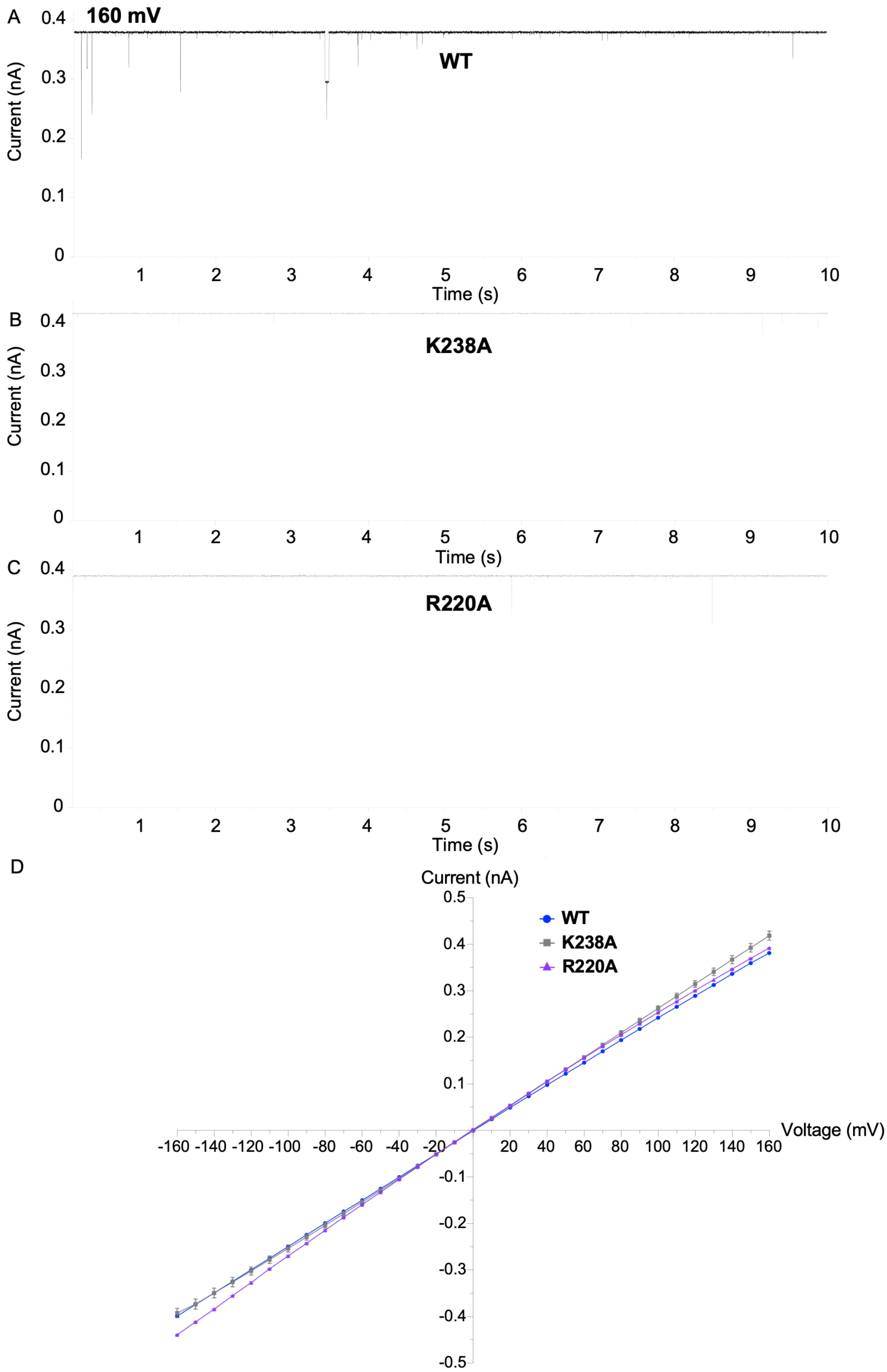

Figure S13. Blank raw current traces at 4 M KCl. Raw current traces at +160 mV of WT **(A)**, K238A **(B)**, R220A **(C)**. IV curves of the corresponding mutants obtained in the range of -160 mV to +160 mV **(D)**; each point is the result of the average of at least n=3 pore replicates.

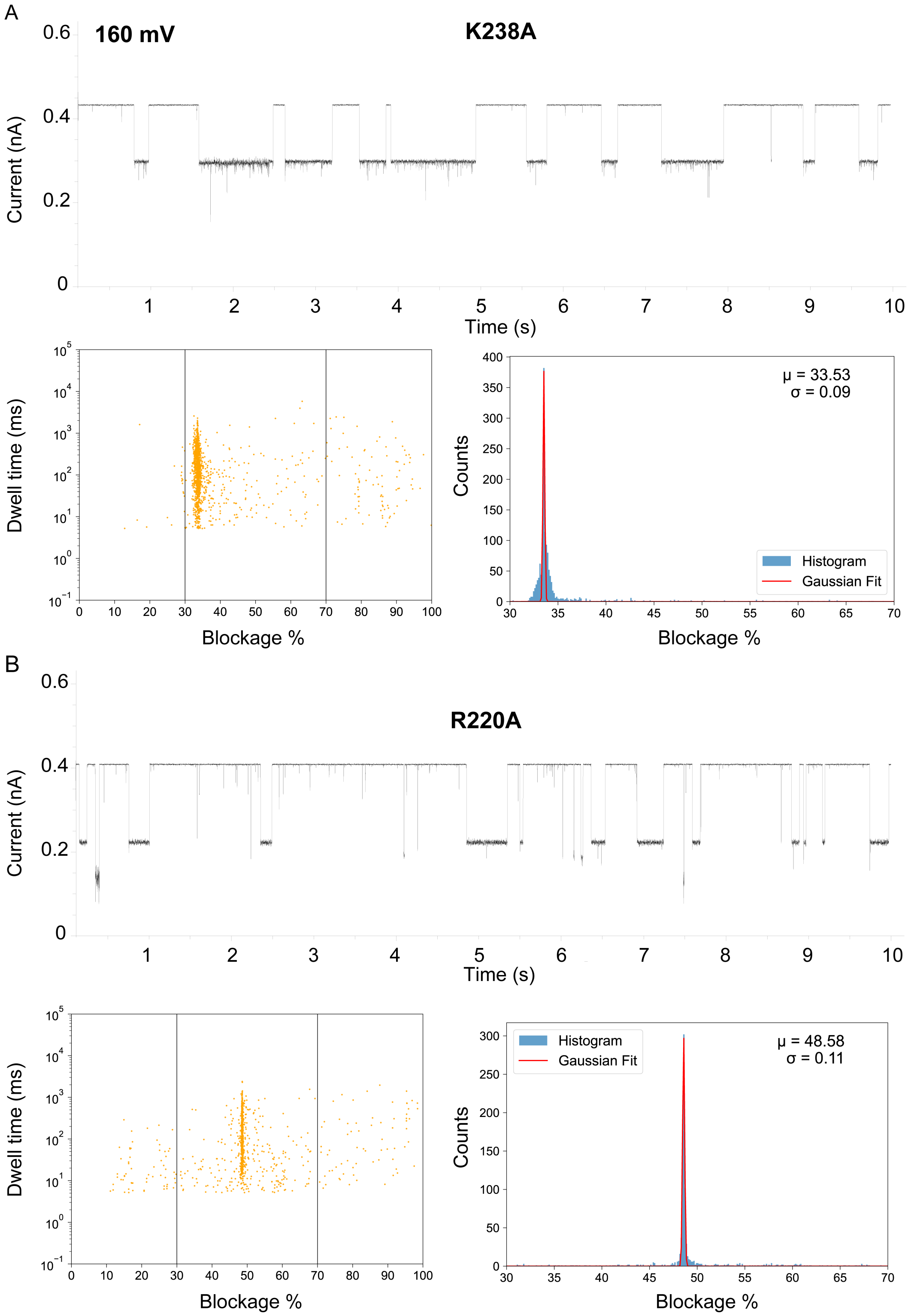

Figure S14. Raw current traces and event selection of MC-LR at +160 mV in AeL mutants. Raw current trace and event selection with K238A (A) and R220A (B). Overlay of scatterplots across at least n=3 pore replicas, event selection denoted by a box (left; 30-70 %), and the respective overlay of current histograms (bin size = 300) produced by the MC-LR interaction with the corresponding AeL mutant (right).

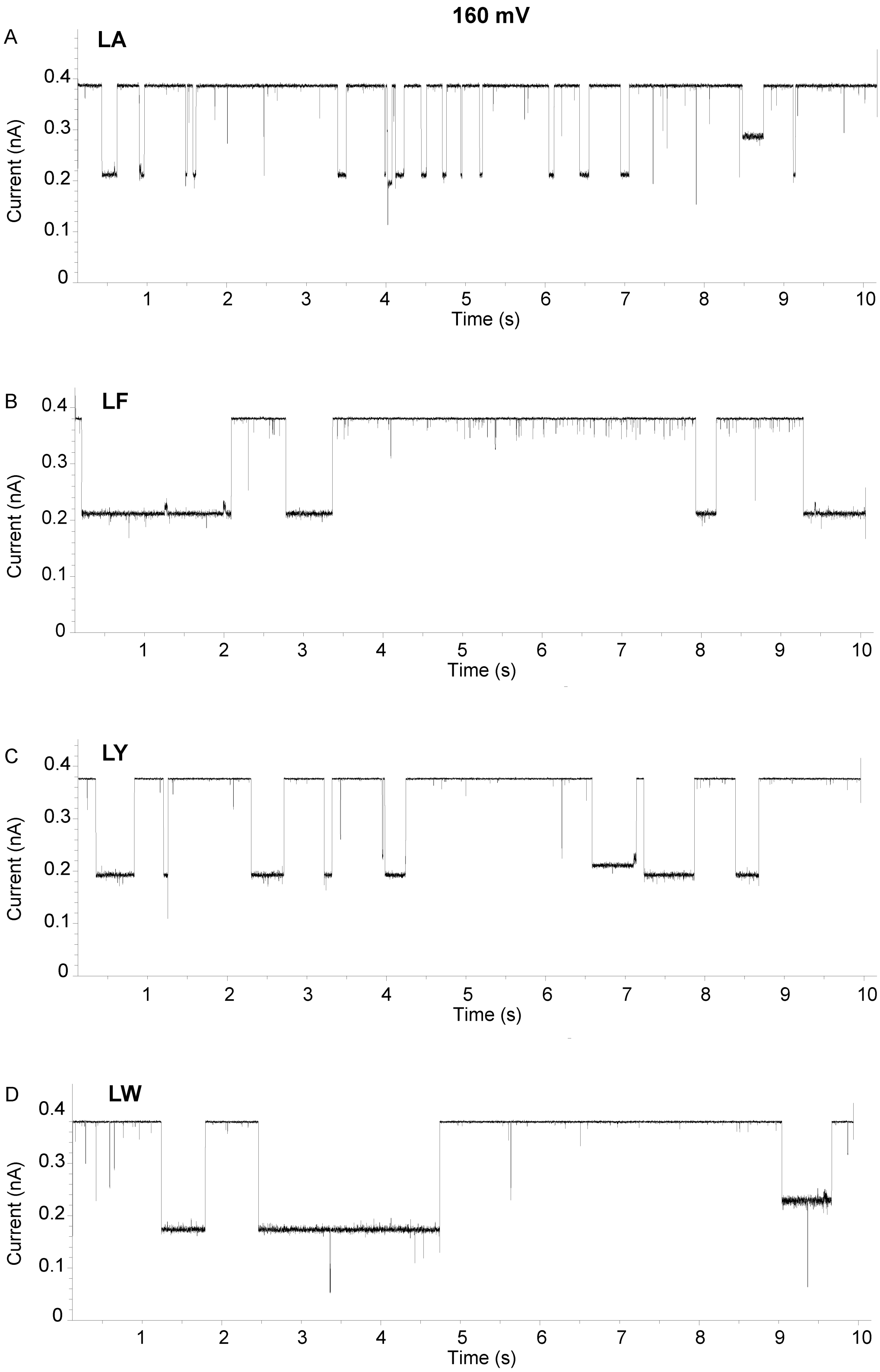

Figure S15. Raw current traces of additional MCs at 160 mV. Raw current traces of MC-LA **(A)**, -LF **(B)**, -LY **(C)**, -LW **(D)** acquired at 160 mV in 4 M KCl, buffered with 10 mM Tris, 1.0 mM EDTA at pH 7.5; traces were filtered to 500 Hz. Data extraction was performed as described in the Methods.

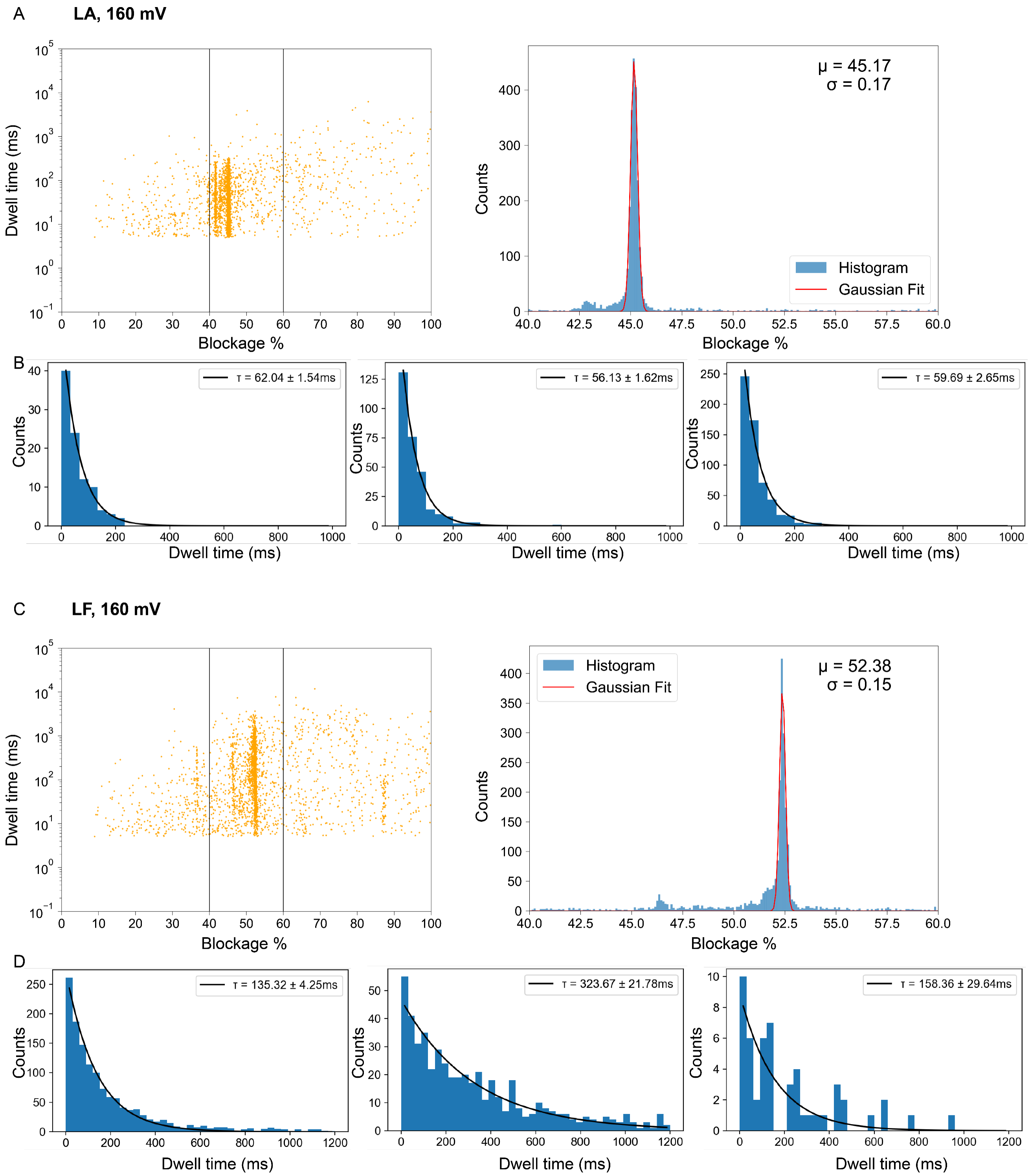

A
LA, 160 mV
Dwell time (ms)
Blockage %
Counts
μ = 45.17
σ = 0.17
Histogram
Gaussian Fit
B
τ = 62.04 ± 1.54ms
τ = 56.13 ± 1.62ms
τ = 59.69 ± 2.65ms
C
LF, 160 mV
μ = 52.38
σ = 0.15
D
τ = 135.32 ± 4.25ms
τ = 323.67 ± 21.78ms
τ = 158.36 ± 29.64ms

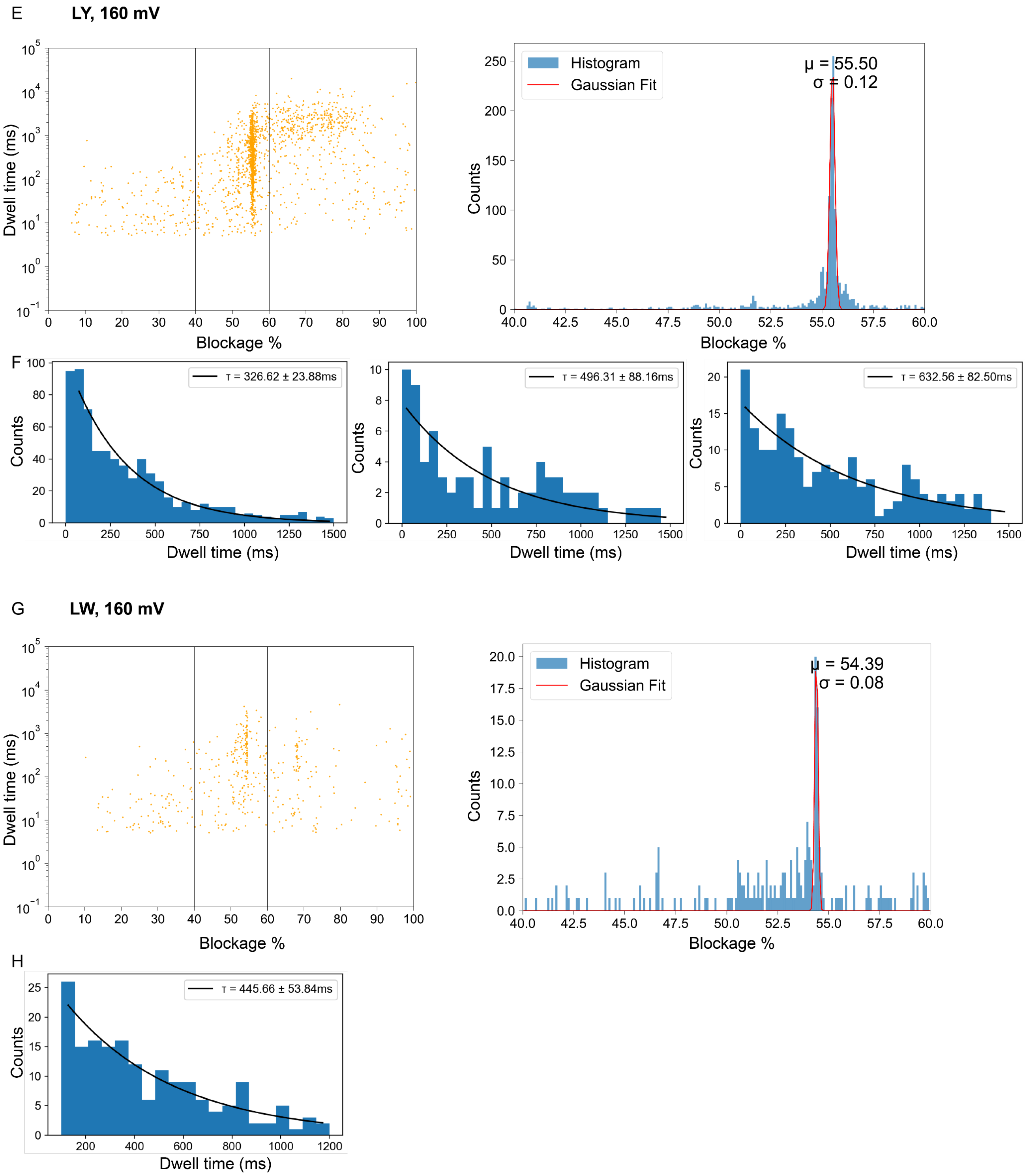

Figure S16. Event selection of MC-LA, -LF, -LY, -LW at 160 mV. (A, C, E, G) Overlay of scatterplots across at least 3 pore replicas, event selection denoted by a box (left; 40-60 %), and the respective overlay of current histograms (bin size = 200) produced by the respective MC interaction with WT AeL (right). **(B, D, F)** Exponential fitting (bin size = 20) of the respective MC dwell times per single pore, with 3 representative individual pore replicas illustrated. **(H)** Exponential fitting of MC-LW dwell times was applied to pooled data from 3 pore replicas to compensate for low event counts per pore. Data extraction was performed as described in the Methods.

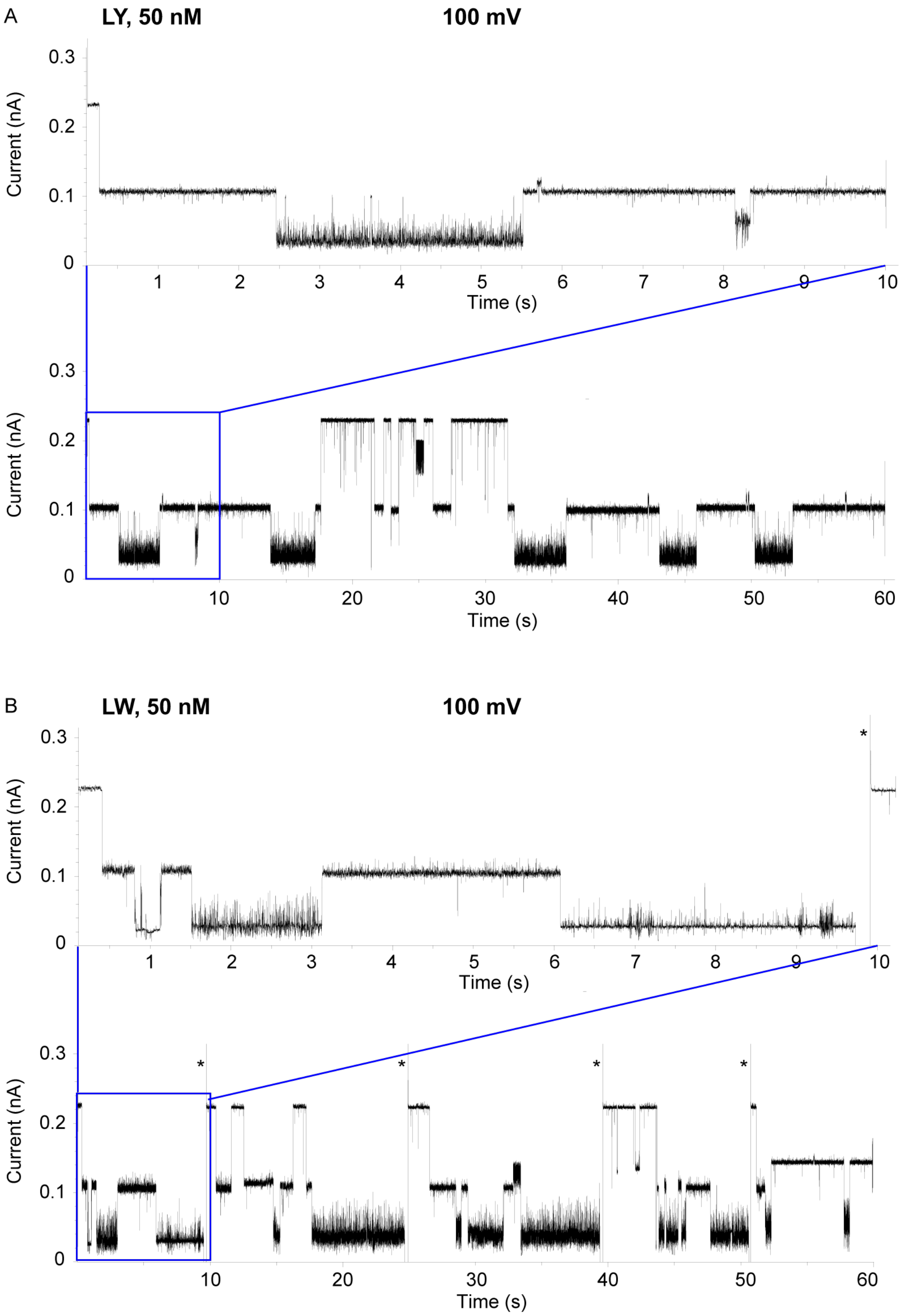

Figure S17. Raw current traces of MC-LY and -LW at 50 nM exhibit frequent two-level blockade events. Recordings were obtained at 100 mV in 4 M KCl, buffered with 10 mM Tris, 1.0 mM EDTA at pH 7.5; traces were filtered to 500 Hz. Panels **A** and **B** show traces for 50 nM MC-LY and 50 nM MC-LW, respectively, with a 10-second (top) and 60-second (bottom) time windows displayed in each panel. Stars (*) depict invert voltage to - 100 mV to unblock the pore, which frequently occurred with MC-LW under these conditions. The second (lower) level may indicate the presence of two MC molecules in the pore—one at the top and one at the bottom of the barrel. A transition from the second to the first level could reflect the upper MC displacing the lower one, initiating a repeating entry–exit cycle.

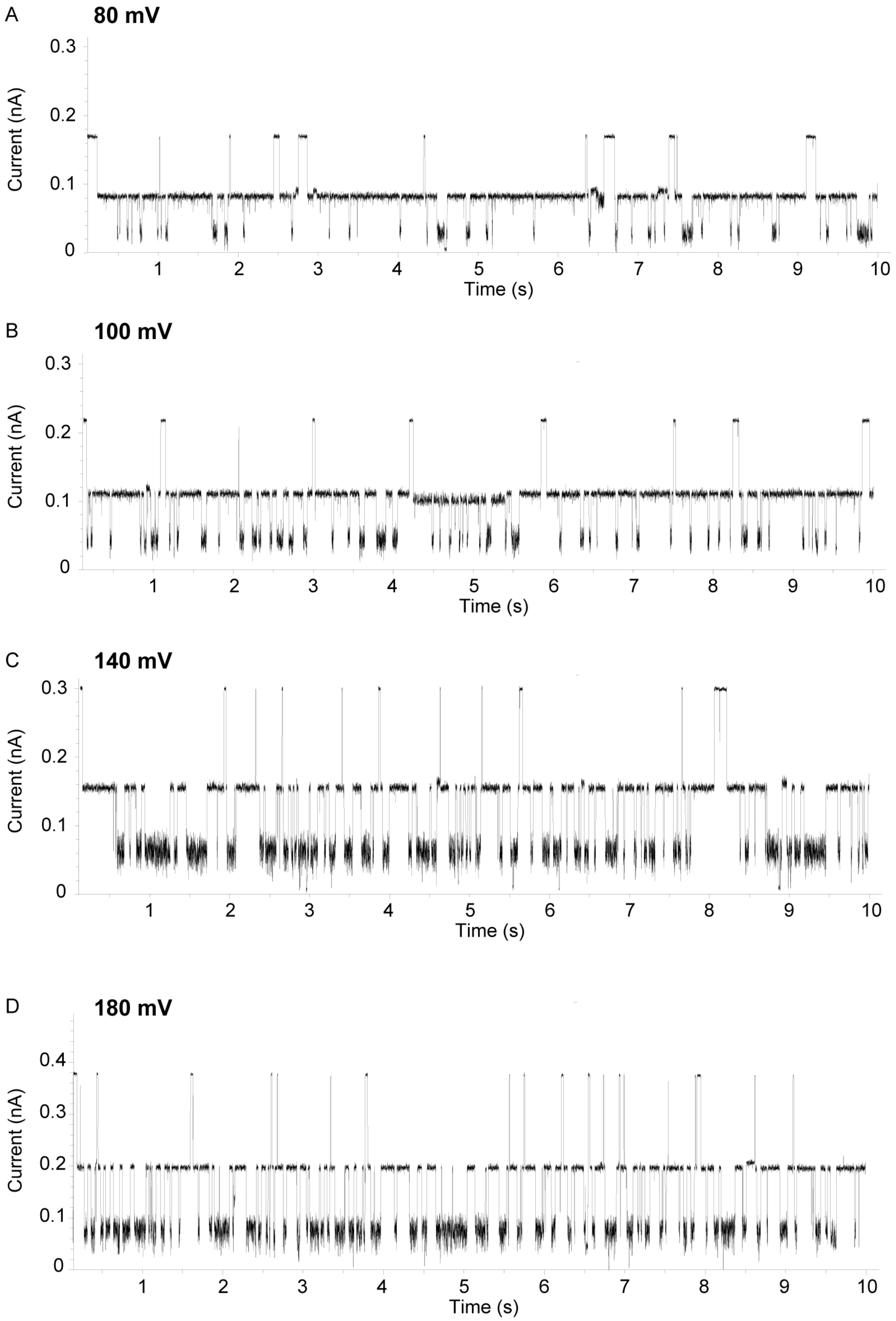

Figure S18. Raw current traces of 2 μM MC-LR exhibit frequent two-level blockade events. Recordings were obtained at 80 mV (**A**), 100 mV (**B**), 140 mV (**C**), 180 mV (**D**) in 4 M KCl, buffered with 10 mM Tris, 1.0 mM EDTA at pH 7.5; traces were filtered to 500 Hz. Voltage increase results in more frequent two-level blockades.

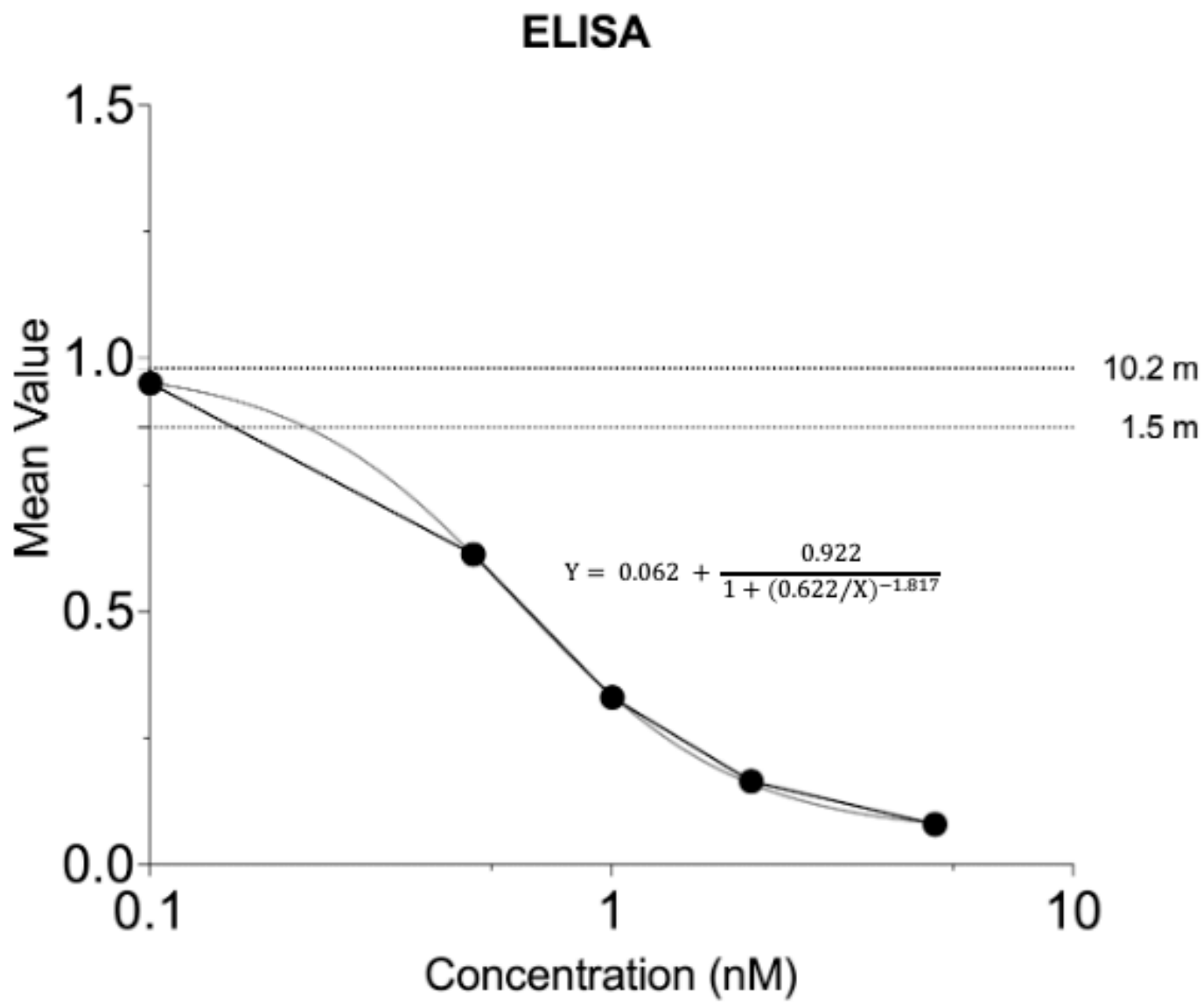

	450 nm	
	Mean OD Value	Conc (nM)
**Sample: 1.5m**	**0.86397**	**0.21839**
NC	1	
C1	0.95101	0.10048
C2	0.61275	0.5024
C3	0.32976	1.0048
C4	0.16457	2.0096
C5	0.07935	5.024
**Sample: 10.2m**	**0.98036**	**0.02825**

Figure S19. ELISA assay on Lake Geneva samples collected at 1.5 m and 10.2 m depths. Samples were analyzed with the Biorbyt ELISA screening kit (UK) per the manufacturer's instructions. The assay is a direct competitive (dcELISA) format: MC toxin in the sample competes with horseradish peroxidase-conjugated MC for a limited amount of antibody coated in the wells. Color development was read as OD at 450 nm using an ELISA reader. Measurements were performed in duplicate, OD of each set of calibrators (C1->C5) and samples were averaged, normalized Mean OD Value calculated as follows: average OD of calibrator or sample/ average OD of negative control (NC). A standard curve of normalized mean OD vs. MC concentration was plotted, and sample MC concentrations were obtained by interpolation.

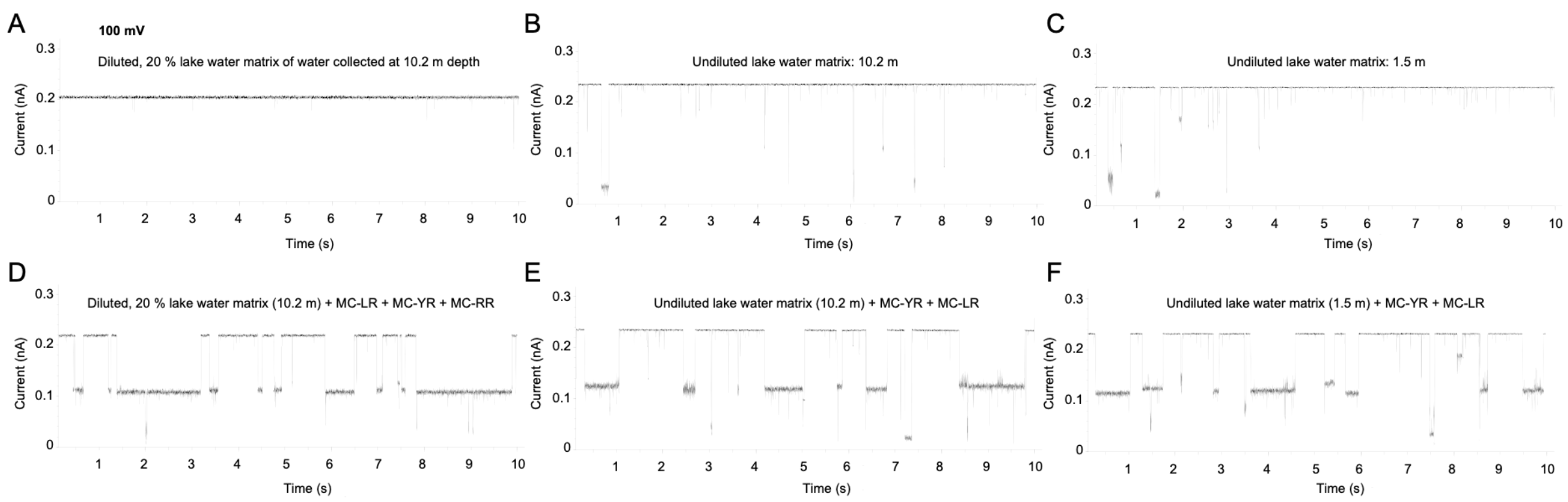

Figure S20. Current traces of the unspiked lake water matrices and MC discrimination in the corresponding matrices (A-C). Background signals from: diluted matrix containing 20 % lake water collected at 10.2 m depth; undiluted lake water collected at 10.2 m depth; undiluted lake water collected at 1.5 m depth. **(D-F)** MC-LR and MC-YR spiking to the respective matrices, yielding the scatterplots and current blockage histograms shown in **Fig. 4**. Note that the spiking experiment in D also included MC-RR, however, for consistency, the MC-RR blockage % region was omitted from **Fig. 4**. Recordings in the unspiked, undiluted matrices revealed noticeable noise originating from the lake-water background, likely due to organic matter, which predominantly caused almost complete pore blockages. Within the expected blockage-percentage window for MC detection, however, no consistent interfering events were observed that would compromise the detection capability.

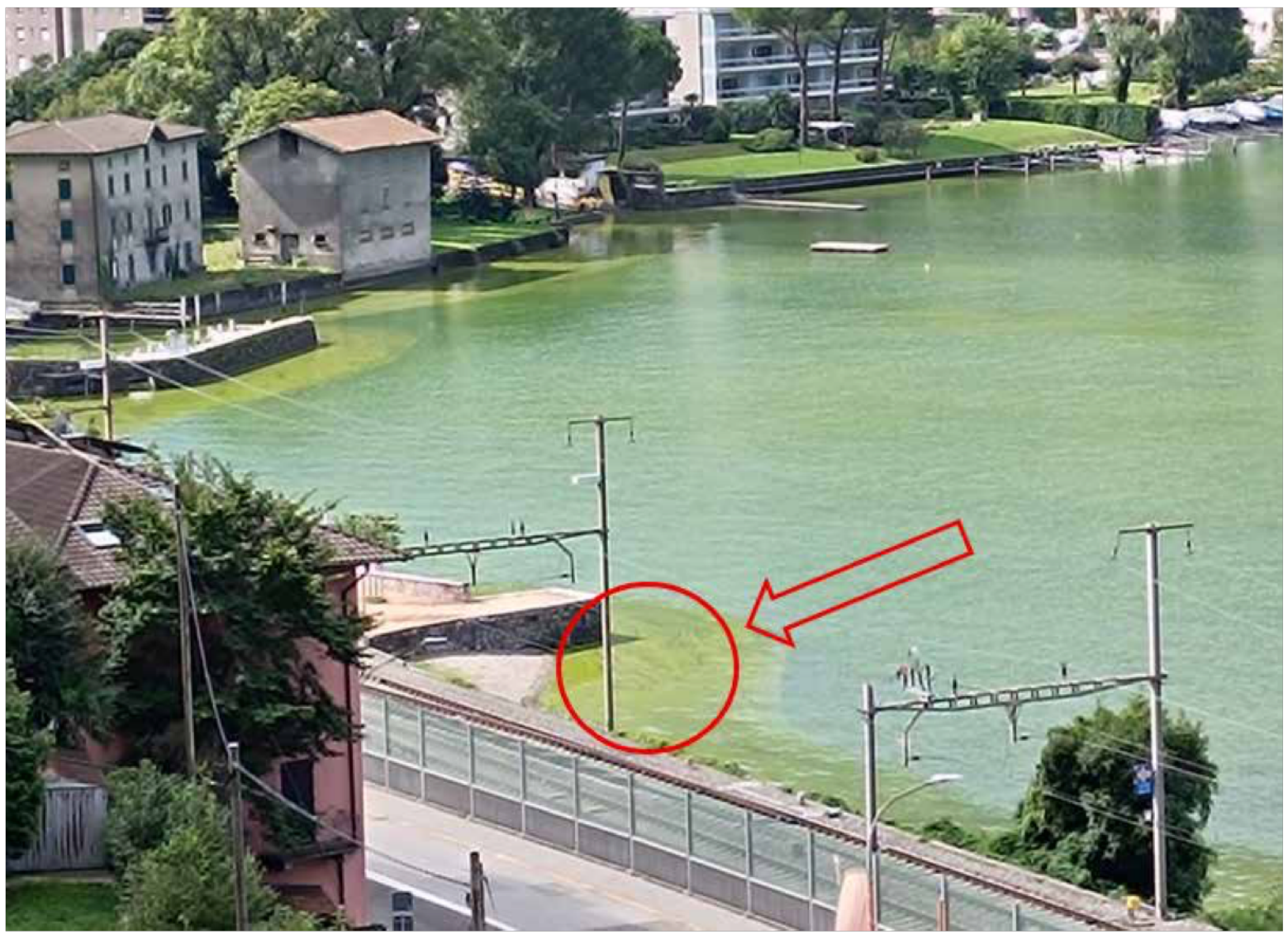

Figure S21. Sampling site at Lake Lugano. Strong cyanobacterial bloom from which the sample was collected. Photo courtesy of Centro di competenze sull'acqua (CCA) SA, Chiasso.

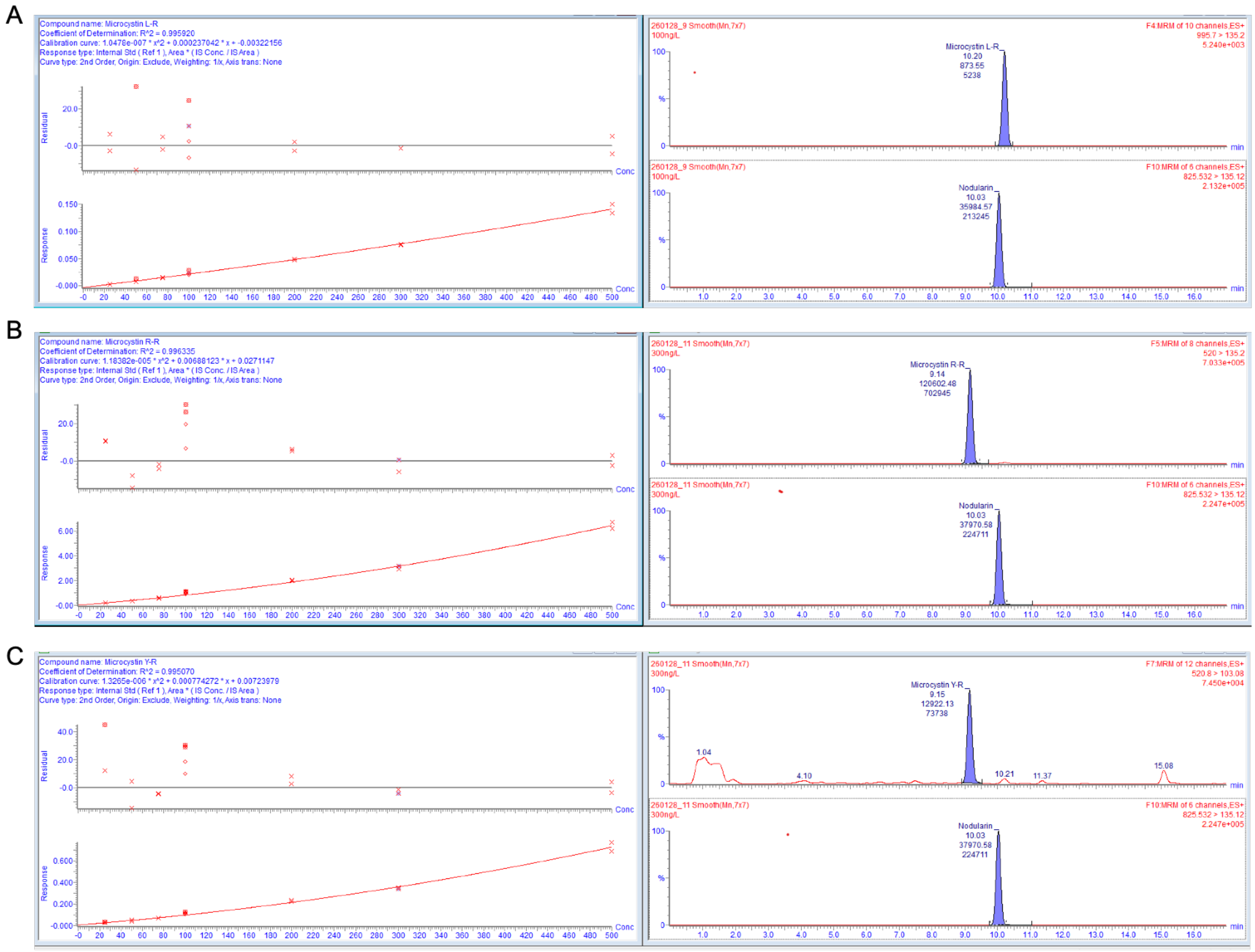

Figure S22. Calibration curves of MC-LR (A), MC-RR (B), and MC-YR (C). Linearity in detector response was observed over the range of 0.025 µg/L to 0.500 µg/L for MC-RR, MC-YR, and MC-LR, yielding R^2 of 0.9963, 0.9951, and 0.9959, respectively.

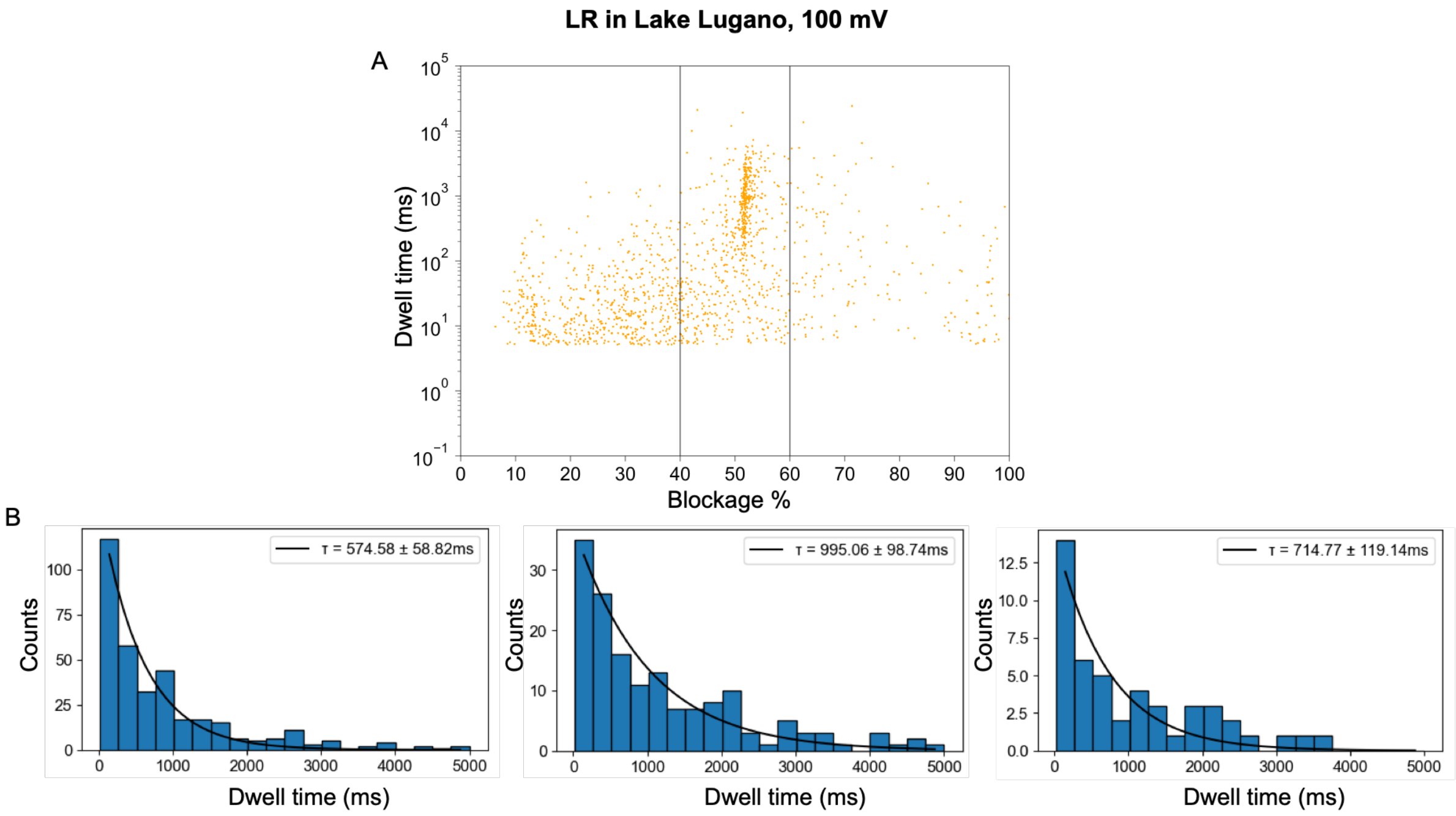

Figure S23. Event selection of MC-LR at 100 mV. (A) Overlay of scatterplots across at least n=3 pore replicas, event selection denoted by a box (40-60 %). **(B)** Exponential fitting (bin size = 20) of the MC-LR dwell times per single pore, with 3 representative individual pore replicates illustrated. Data extraction was performed as described in the Methods.

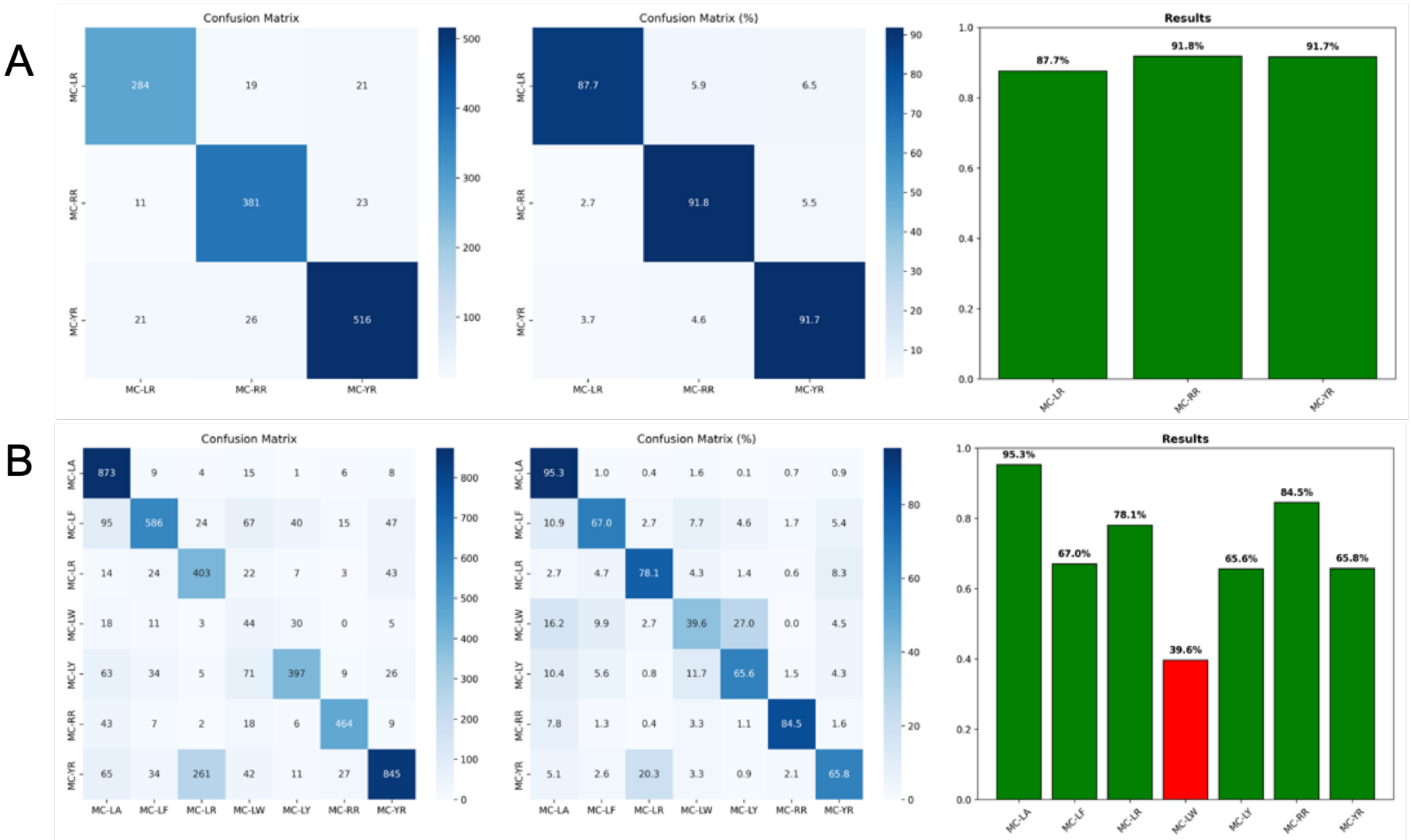

Fig. S24. LSTM of MC-LR, -RR; -YR at 100 mV and MC-LR, -RR; -YR, -LA, -LF, -LY, -LW at 160 mV. (A, B) Confusion matrix and prediction accuracy for 3 MCs and 7 MCs, respectively. Event traces were truncated or zero-padded to a fixed length and divided into N segments, from which 14-dimensional feature vectors (mean, standard deviation, minimum, maximum, skewness, kurtosis, threshold-crossing count, 10–90% amplitude range, spectral centroid, relative segment duration, and four FFT-derived spectral descriptors) were extracted per segment and normalized. These feature sequences (N×14) were used to train a bidirectional LSTM with an attention layer and fully connected classifier, optimized with weighted cross-entropy (class weights from inverse label frequencies) and AdamW, and evaluated using stratified train/test splits with early stopping based on validation loss.

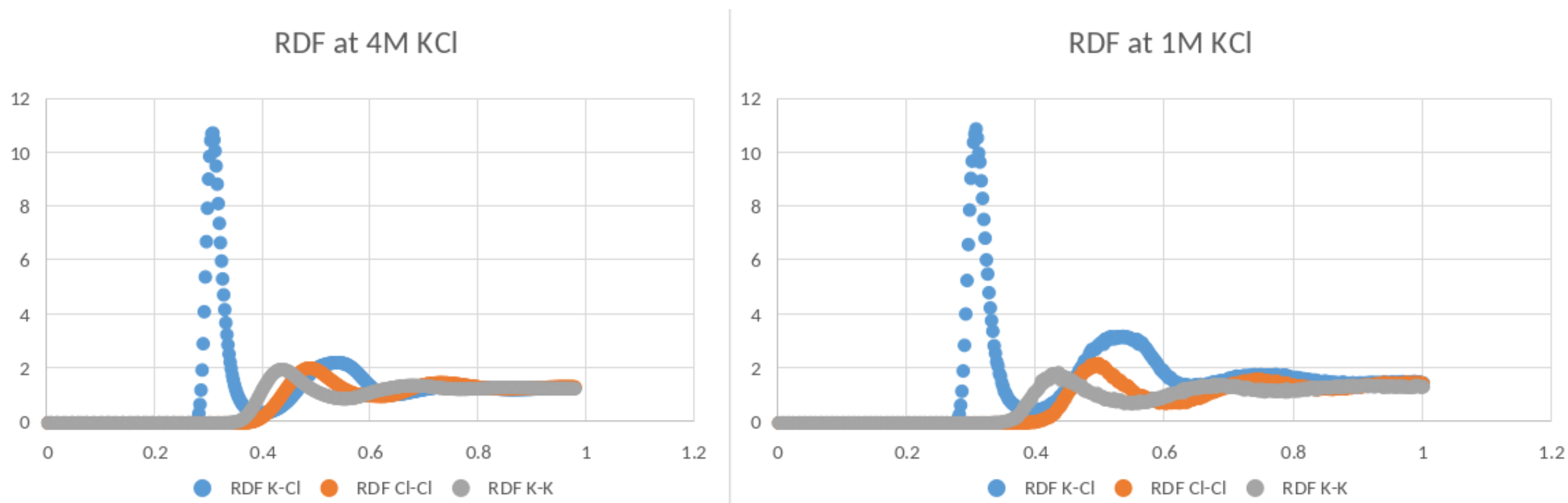

Fig. S25. Radial distribution function (RDF) between K^+ and Cl^- (blue), K^+ and K^+ (grey), Cl^- and Cl^- (orange) at 4 M KCl (left) and 1 M KCl (right). The results show no significant difference between the two simulations, indicating no artificial aggregation due to any saturation.

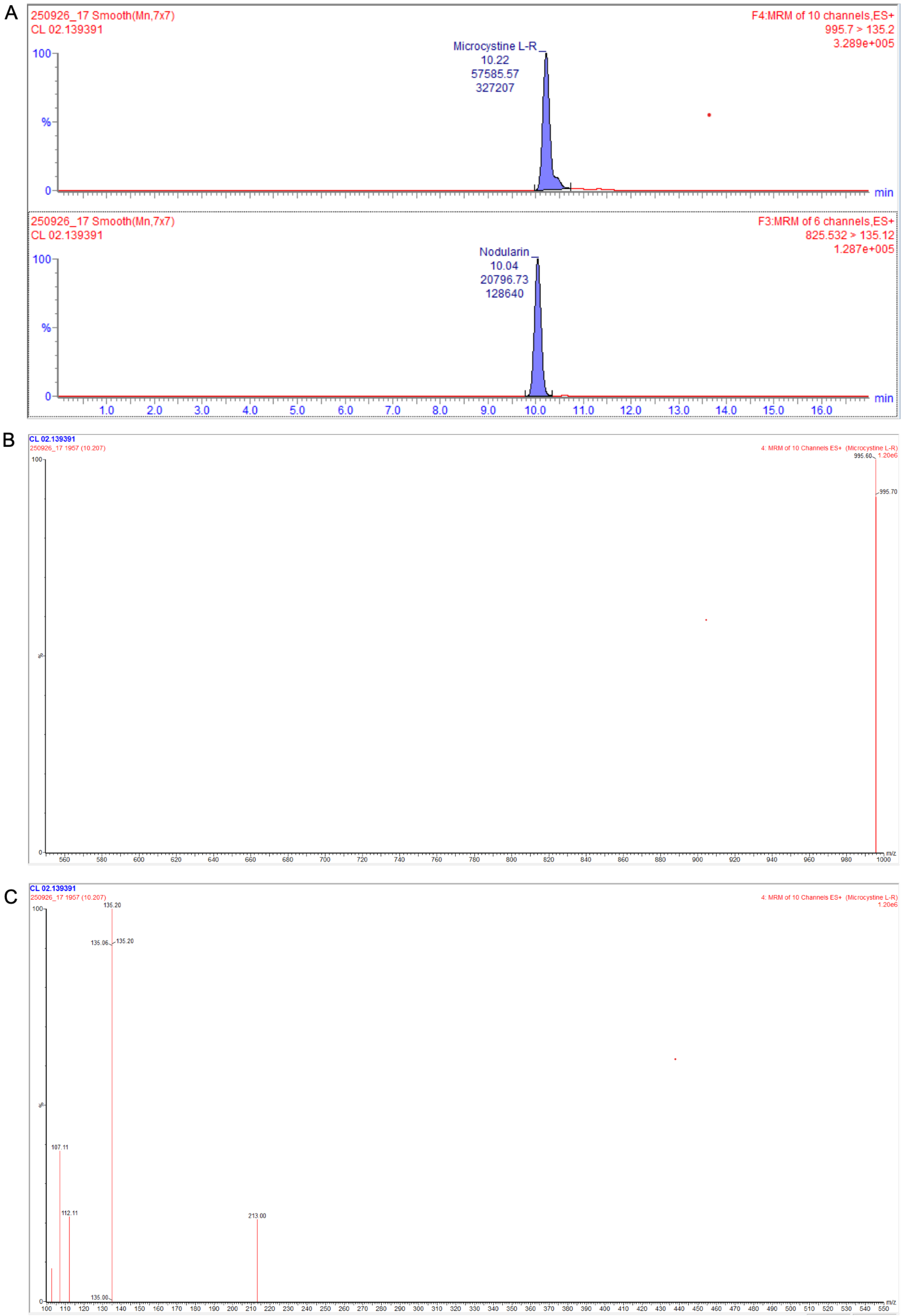

Figure S26. LC-MS/MS of the Lake Lugano sample. (A) Chromatogram of the sample. (B) Mass spectrum showing the MC-LR parent ion at m/z 996. (C) MS/MS spectrum showing the corresponding product ion at m/z 135.

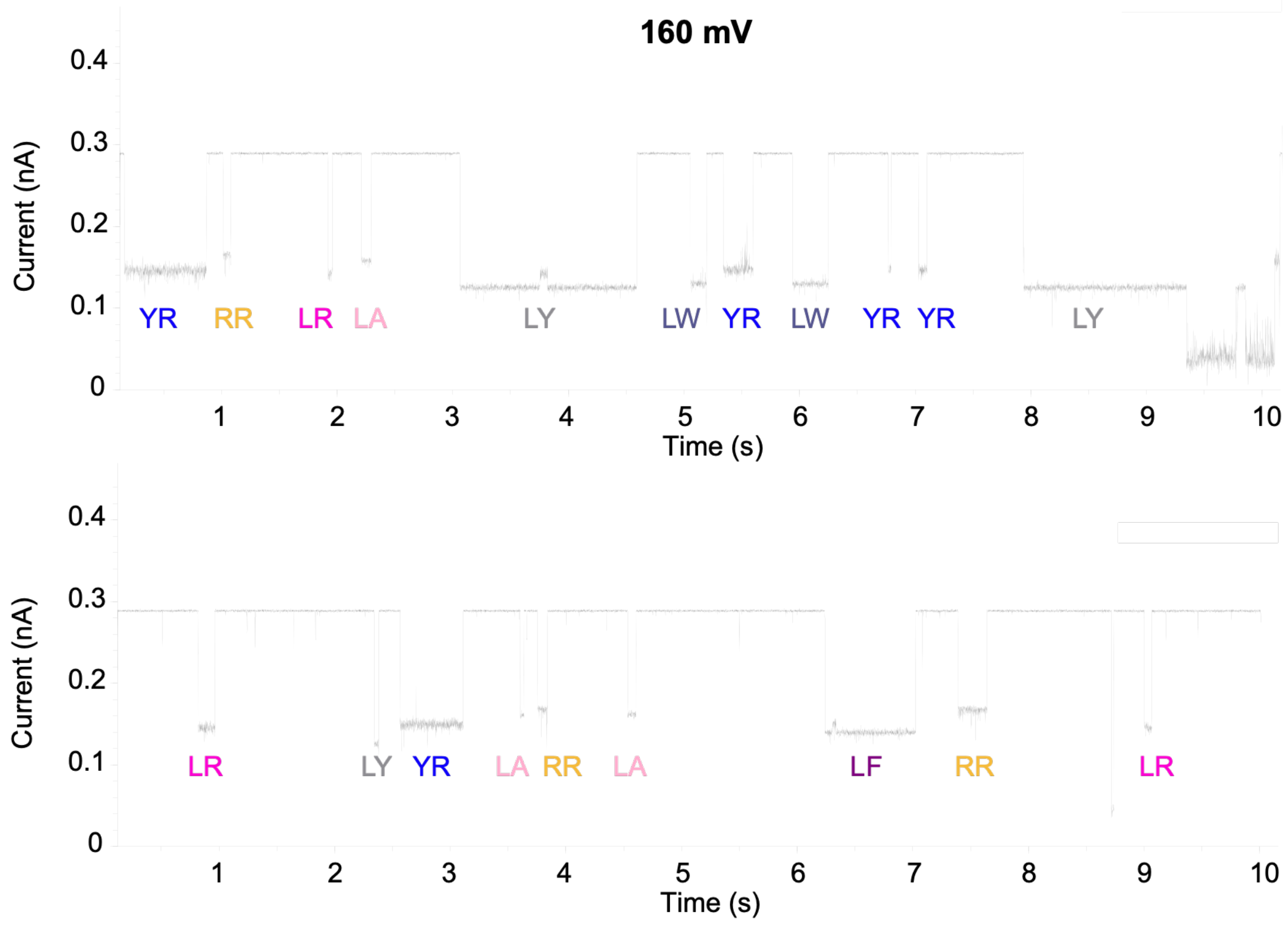

Figure S27. Raw current trace for simultaneous MCs detection. Raw current trace of a 7 MC mixture experiment. Representative trace segments recorded at 160 mV in 4 M KCl, buffered with 10 mM Tris and 1.0 mM EDTA at pH 7.5, and low-pass filtered to 500 Hz. Two 10 s excerpts from a 35 min single-pore recording are shown, highlighting events corresponding to seven annotated MC congeners. The full 35 min recording was used to generate the scatterplot and histogram presented in **Fig. 3C**. Data extraction was performed as described in the Methods.

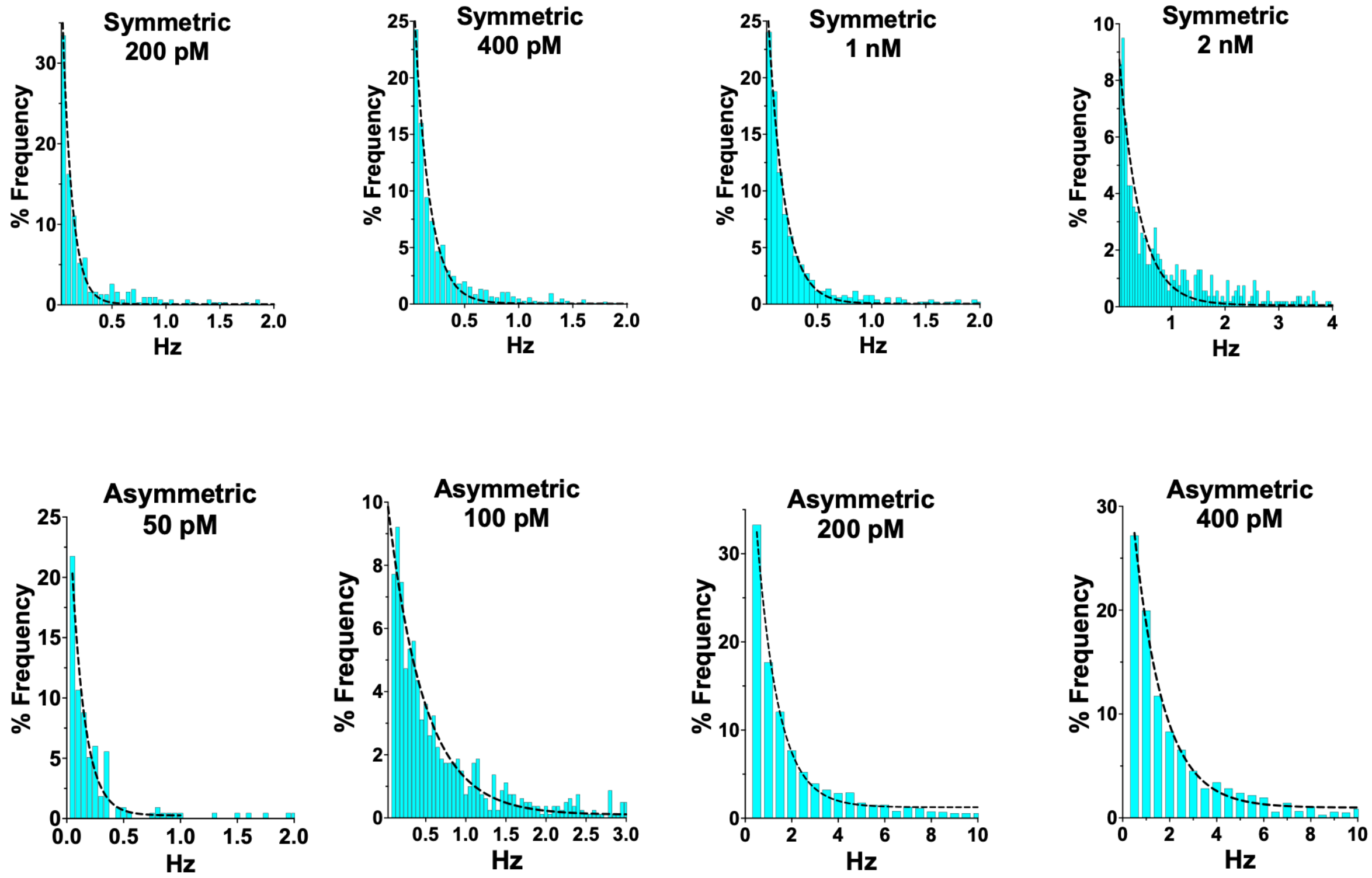

Figure S28 Frequency plots for symmetric and asymmetric conditions. From these plots, the frequency of events was extracted and plotted to generate **Figure 6C**.

Supplementary Tables

Table S1. Guideline values for MC-LR and exposure scenarios[4].

Toxin	Exposure	Value type (*μg/L*)
Microcystin-LR	Drinking-water, lifetime	1
Microcystin-LR	Drinking-water, short term	12
Microcystin-LR	Recreational	24

Table S2. Summary of physical and chemical properties of the tested MC congeners[5]. Weight and volume of the variable amino acid, weight and net charge of the respective microcystin congener.

Amino acid X (position 2)	Weight (Da)	Volume (Å^3)	MCXR (Da)	MCXR Net charge
L	131	166.7	995.2	-1
R	174	173.4	1038.2	0
Y	181	193.6	1045.2	-1

Amino acid Z (position 4)	Weight (Da)	Volume (Å^3)	MCLZ (Da)	MCLZ Net charge
A	89	88.6	910	-2
F	165	189.9	986.2	-2
Y	181	193.6	1002.2	-2
W	204	227.8	1025.2	-2

Table S3. Analysis of $^3J_{HN\text{-}HA}$ coupling constants and $^{13}C\alpha$ and $^{13}C\beta$ chemical shifts for relevant MCLR and MCRR.
Nres indicates residue number, with the canonical residue names shown on the first column. Cα and Cβ refer to the chemical shifts of ^{13}C nuclei in the MC backbones, akin to a small peptide (Cα on the cycle, Cβ first C atom in the sidechain). These chemical shifts reflect local conformational dynamics, interpreted as for peptide/protein chemical shifts by calculating $\Delta\delta_{C\alpha} - \Delta\delta_{C\beta}$, a calibration-insensitive probe that deviates from zero for structured regions, with higher values indicating stronger structuring. Similarly, $^3J_{HN\text{-}HA}$ reflects conformational averaging of the H-N-CA-HA dihedral, although here their differences are not too informative. In bold we highlight the key values discussed.

	MC-LR							MC-RR							
**Residue**	**Nres**	**$\delta_{C\alpha}$**	**$\delta_{C\beta}$**	**$\delta_{C\alpha}-\delta_{C\alpha rc}$***	**$\delta_{C\beta}-\delta_{C\beta rc}$***	**$\Delta\delta_{C\alpha}-\Delta\delta_{C\beta}$**	**$^3J_{HN\text{-}HA}$ (Hz)**	**Nres**	**$\delta_{C\alpha}$**	**$\delta_{C\beta}$**	**$\delta_{C\alpha}-\delta_{C\alpha rc}$***	**$\delta_{C\beta}-\delta_{C\beta rc}$***	**$\Delta\delta_{C\alpha}-\Delta\delta_{C\beta}$**	**$^3J_{HN\text{-}HA}$ (Hz)**	**ΔJ (Hz)**
Ala	1	49.54	15.92	-2.961	-3.08	0.121	6.92	1	50.01	15.94	-2.491	-3.06	0.57	6.44	0.48
Leu/Arg	2	53.59	39.22	-2.114	-2.68	**0.569**	7.27	2	55.56	28.2	-0.743	-2.1	**1.361**	6.62	0.65
Masp	3	55.99	40.87				9.8	3	56.33	41.15				9.98	-0.2
Arg	4	51.68	27.22	-4.624	-3.08	-1.54	9.8	4	51.59	27.33	-4.708	-2.97	-1.74	9.92	-0.1
ADDA	5	55.26	43.93				9.2	5	55.5	43.82				9.6	-0.4
Glu	6	54.76	26.32	-1.936	-3.38	**1.444**	6.95	6	55.93	26.23	-0.768	-3.47	**2.701**	6.1	0.85
MDHA	7		115.9					7		115.1					

*$\delta_{C\alpha rc}$ and $\delta_{C\beta rc}$ are the random coil chemical shifts tabulated for each amino acid in [6].

Table S4. Concentration-dependent event frequency (Hz) of MC-LR events (Fig. 6C).

**Asymmetric (averaged over pores)**		
**x: Concentration (nM)**	**y: Frequency (Hz)**	**Standard deviation**
0.025	0.119	0.0605
0.05	0.135	0.0247
0.1	0.3016	0.104
0.2	0.595	0.132
0.4	0.94	0.0694
**Symmetric (averaged over pores)**		
**x: Concentration (nM)**	**y: Frequency (Hz)**	**Standard deviation**
0.2	0.05547871	0.00982665
0.4	0.07616169	0.01316161
1	0.08090074	0.02409083
2	0.1861599	0.06369867
**Lake Lugano (averaged over pores)**		
**x: Concentration (nM)**	**y: Frequency (Hz)**	**Standard deviation**
From the event frequency equation for symmetric condition: **x**=(y-0.03725)/0.06936= **12.73111449 ± 2.155575193**	0.920280101	0.149510695

Table S5. LC-MS/MS analysis of the Lake Lugano sample.

	**MC-LR [µg/L]**
**Method**	LC-MS/MS
Sample : Lake Lugano	13.038 ± 2.61

Table S6. LC Gradient used for LC-MS/MS analysis.

**Time (min)**	**Flow (ml/min)**	**%A**	**%B**	**Curve**
Initial	0.34	100	0	Initial
1	0.34	100	0	6
11.3	0.34	0	100	6
13	0.34	0	100	6
17	0.34	100	0	1

Table S7. MRM transition of ESI.

**Name**	**Parent (m/z)**	**Daughter (m/z)**
Nodularin	825.53	135.12
MC-RR	520.00	135.20
MC-LR	995.70	135.20
MC-YR	520.8	103.08

References

1. Harada, K. ichi *et al.* Isolation and characterization of the minor components associated with microcystins LR and RR in the cyanobacterium (blue-green algae). *Toxicon* **28**, 55–64 (1990).
2. Harada, K. I. *et al.* Structural determination of geometrical isomers of microcystins lr and rr from cyanobacteria by two-dimensional nmr spectroscopic techniques. *Chem. Res. Toxicol.* **3**, 473–481 (1990).
3. Trogen, G. B. *et al.* Conformational studies of microcystin-LR using NMR spectroscopy and molecular dynamics calculations. *Biochemistry* **35**, 3197–3205 (1996).
4. Chorus, I. & Welker, M. *Toxic Cyanobacteria in Water*. *Toxic Cyanobacteria in Water* (CRC Press, 2021). doi:10.1201/9781003081449.
5. Abriata, L. A., Merijn, M. L. & Tomatis, P. E. Sequence-function-stability relationships in proteins from datasets of functionally annotated variants: The case of TEM β-lactamases. *FEBS Lett.* **586**, 3330–3335 (2012).
6. Berjanskii, M. V & Wishart, D. S. Application of the random coil index to studying protein flexibility. *J. Biomol. NMR* **40**, 31–48 (2008).
